# Epigenetic and transcriptional regulations prime cell fate before division during human pluripotent stem cell differentiation

**DOI:** 10.1038/s41467-023-36116-9

**Published:** 2023-01-25

**Authors:** Pedro Madrigal, Siwei Deng, Yuliang Feng, Stefania Militi, Kim Jee Goh, Reshma Nibhani, Rodrigo Grandy, Anna Osnato, Daniel Ortmann, Stephanie Brown, Siim Pauklin

**Affiliations:** 1 Department of Surgery, University of Cambridge, Cambridge, CB2 0QQ UK; 2 Wellcome Sanger Institute, Wellcome Genome Campus, Hinxton, CB10 1SA UK; 3 Wellcome – MRC Cambridge Stem Cell Institute, University of Cambridge, Cambridge, CB2 0SZ UK; 4 Botnar Research Centre, Nuffield Department of Orthopaedics, Rheumatology and Musculoskeletal Sciences, Old Road, University of Oxford, Headington, Oxford, OX3 7LD UK; 5 Present Address: European Molecular Biology Laboratory, European Bioinformatics Institute, Wellcome Genome Campus, Hinxton, CB10 1SD UK; 6 Present Address: The Francis Crick Institute, London, NW1 1AT UK

**Keywords:** Stem-cell differentiation, Epigenetics, Pluripotent stem cells, Gene regulatory networks, Differentiation

## Abstract

Stem cells undergo cellular division during their differentiation to produce daughter cells with a new cellular identity. However, the epigenetic events and molecular mechanisms occurring between consecutive cell divisions have been insufficiently studied due to technical limitations. Here, using the FUCCI reporter we developed a cell-cycle synchronised human pluripotent stem cell (hPSC) differentiation system for uncovering epigenome and transcriptome dynamics during the first two divisions leading to definitive endoderm. We observed that transcription of key differentiation markers occurs before cell division, while chromatin accessibility analyses revealed the early inhibition of alternative cell fates. We found that Activator protein-1 members controlled by p38/MAPK signalling are necessary for inducing endoderm while blocking cell fate shifting toward mesoderm, and that enhancers are rapidly established and decommissioned between different cell divisions. Our study has practical biomedical utility for producing hPSC-derived patient-specific cell types since p38/MAPK induction increased the differentiation efficiency of insulin-producing pancreatic beta-cells.

## Abstract (web summary)

Many stem cells exhibit cell division coupled to differentiation, though the changes occurring between consecutive cell divisions have been difficult to study. Here they use synchronized hPSC culture to show that production of transcription factors and epigenetic changes are linked with cell division timing.

## Introduction

Epigenome mapping during cellular differentiation has been an area of great interest in recent years^1^. Histone modifications, chromatin organisation and regulatory regions such as enhancers have been directly linked to the establishment of cellular identity during differentiation of embryonic and somatic stem cells^2–4^. However, these studies have often compared undifferentiated cells with cells produced several days after induction of differentiation, thereby excluding early events inducing the acquisition of new cellular identities^1,5–7^. Furthermore, differentiation is often, if not systematically, associated with cell division in mammalian stem cells^8^. Consequently, mechanisms directing the acquisition of a new cellular identity are likely to be dynamically regulated between successive cell divisions. However, the study of these cell cycle-related mechanisms is technically challenging in vivo due to ethical considerations, especially in humans, the quantity of biological material, and the complexity of the cellular microenvironment. In addition, stem cells grown in vitro are often heterogeneous in nature which renders difficult the investigation of cell cycle-related mechanisms during differentiation. Together, these limitations have concealed dynamic epigenetic regulations during cell divisions upon differentiation. Human Pluripotent Stem Cells (hPSCs) make it possible to partly address these questions since hPSCs can be grown in vitro almost indefinitely while maintaining their capacity to differentiate into a near homogenous population of primary germ layer progenitors. For that, defined culture conditions devoid of unknown factors interfering with the experimental outcome can be used^9^. Furthermore, previous studies have shown or suggested that transcription factors, epigenetic modifiers, and signalling pathways are able to control cell fate choices in hPSCs^10^. Accordingly, enhancer maps have been established very precisely during differentiation of hPSCs^1,3^, while the functions of transcription factors directing endoderm differentiation, such as EOMES, GATA6, SOX17 and FOXA2 have been identified^11–15^. Finally, we and others have shown that human embryonic stem cells (hESCs) can be synchronised in different phases of their cell cycle using the fluorescent ubiquitination-based cell cycle indicator (FUCCI) reporter system, and then induced to differentiate into a near homogeneous population of endoderm cells^16–18^.

Here, we take advantage of the hESC-FUCCI reporter and further develop a culture system to differentiate hESCs synchronised in the early G1 phase of their cell cycle. This approach enabled us to analyse epigenetic modifications occurring during the two first cell divisions leading to endoderm, uncovering the early changes in the epigenome that direct the acquisition of a new cellular identity during differentiation.

## Results

### Culture system to differentiate synchronised stem cells

**Fig. 1:**
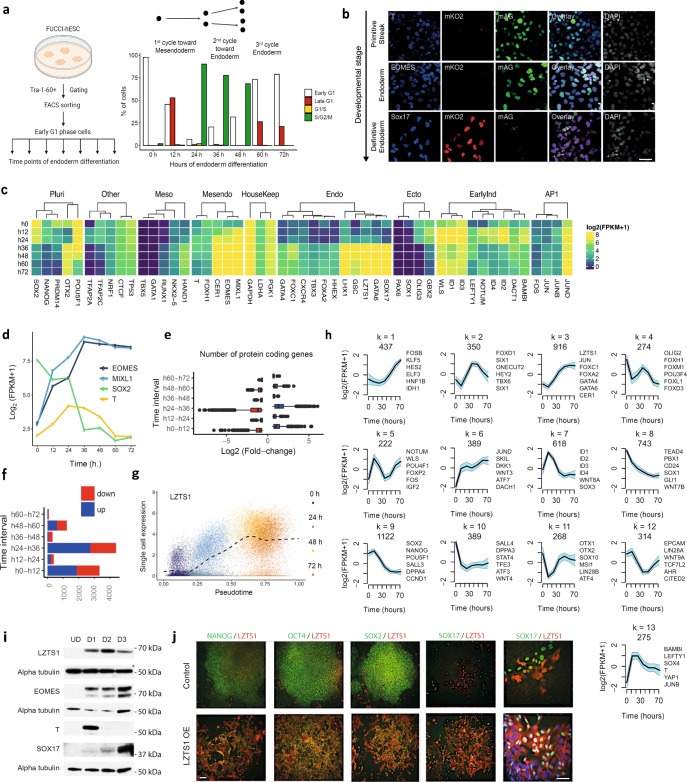
Differentiation of cell cycle synchronised hESCs reveals that each cell division results in a new cellular identity. **a** Schematic representation of experimental setup to differentiate synchronised EG1-FUCCI hPSCs into endoderm. The bar plot indicates the proportion of cells in the population at each cell cycle phase and time point. The schematic representation was created with BioRender.com. **b** Immunofluorescence analyses showing expression of the primitive streak (T, 36 h), early-endoderm (EOMES, 48 h) and definitive endoderm (SOX17, 72 h) markers in FUCCI-hESCs differentiating into endoderm after synchronisation. For immunostaining, the scale bar represents 100 µm. **c** Gene expression analyses show stage-specific and selected genes that are differentially expressed upon differentiation. **d** Mean gene expression of *T*, *EOMES*, *MIXL1* and *SOX2* during the time course. **e** The number of differentially expressed genes (FC >1.5; FDR ≤0.01), and **f** distribution of log_2_ fold changes during endoderm differentiation. In (**e**), the boxes show the interquartile range, with the median marked as a heavy vertical band. Whiskers represent the highest (lowest) datapoint within 1.5 times the interquartile range of the 75th (25th) percentile. Outliers are plotted separately as individual points beyond the whiskers on the boxplot. Data in (**e**) depicts *n* = 3 biologically independent samples over an independent experiment. **g** Single-cell expression (*y*-axis) of *LZTS1*, plotted along pseudotime (*x*-axis). The black dashed line indicates the smoothed mean. Gene expression values correspond to log_2_(CPM + 1). **h** Average pattern of gene expression of the 13 clusters identified by *k*-means clustering. Representative genes are shown beside each cluster. The *y*-axis represents row-scaled log_2_(FPKM + 1). The interval represents two standard deviations from the mean. **i** Western blot analyses show the expression of mesendoderm markers (T), endoderm markers (SOX17, EOMES) and LZTS1 during the differentiation of hPSCs into endoderm. **j** Immunostaining showing the expression of pluripotency markers (NANOG, OCT4 and SOX2), endoderm markers (SOX17) and LZTS1 in hPSCs (control) and hPSCs overexpressing LZTS1 (LZTS1 OE). For immunostaining, the scale bars represent 100 µm. Source data are provided as a Source Data file.

We and others have shown that hESCs can only be induced to differentiate into endoderm during the G1 phase of their cell cycle^16,18^. Thus, we hypothesised that hESCs synchronised in G1 could differentiate homogenously while progressing through their cell cycle. To confirm this possibility, a quasi-homogenous population of hESCs was isolated in the early G1 phase (EG1-hPSCs) by cell sorting using the FUCCI reporter system (Fig. 1a; Supplementary Fig. 1a; gating strategy shown in Supplementary Fig. 7a)^18,19^. FUCCI is a two-colour (red and green) indicator that enables to track cell cycle progression in live cells without the need for chemical inhibitors that could perturb stem cell characteristics^16,18^. The sorted cells were then replated in culture conditions inductive for endoderm differentiation following a protocol previously validated in a diversity of hPSC lines^20,21^. The resulting cells differentiated into a population of mesendoderm cells expressing the protein T after 36 h, and definitive endoderm cells expressing the protein SOX17 after 48 h (Fig. 1b). Further analyses revealed that EG1-hPSCs progressed through differentiation while being synchronised for their cell cycle for 24 h which corresponds to the duration of the first cell cycle after induction of differentiation (Supplementary Fig. 1a)^22^. However, the cell cycle synchronisation was progressively lost between 24 and 36 h. In other words, EG1-hPSCs progressed through the S phase after 12 h and underwent a first division after 24 h to re-enter the S phase 36 h after the induction of differentiation. The resulting mesendoderm cells underwent a second division around 48 h to become endoderm cells, remaining blocked in G1 until the end of our experimental time frame (72 h) (Fig. 1a and Supplementary Fig. 1a). The precise reason for the loss of synchronisation is not known but a similar reduction of cell synchronisation is observed also in other cell systems. The gradual loss of synchronisation could be caused by multiple effects, such as spontaneous DNA lesions that arise in individual cells and will slightly impact the speed of DNA replication, transient activation of DNA damage checkpoints or other cell cycle phases such as spindle checkpoint in M phase. Over time these stochastic molecular events will decrease the synchronised cell cycle progression of cells.

Our past research has shown that early G1 phase cells seem to be particularly ready to undergo endoderm differentiation since SMAD2/3 can bind to several endoderm loci, such as MIXL1 and SOX17, in undifferentiated hPSCs^18^. To further answer the question if the early G1 phase has higher expression of certain transcription factors compared to other cells in the late G1 or S/G2/M phase on day 0 itself, we looked at the expression of endoderm factors in different cell cycle phases of pluripotent undifferentiated cells by using FACS sorting followed by qPCR (Supplementary Fig. 1b). Our results revealed that some of the key endodermal genes such as *MIXL1*, *EOMES*, *GATA4* and *SOX17* have an elevated transient expression in the early G1 phase of day 0 undifferentiated hPSCs compared to other cell cycle phases. The cells in the early G1 phase rapidly initiated endoderm differentiation as shown by the relative increase in activating histone mark H3K27ac analysed by ChIP-qPCR of genes with different expression dynamics (*ID1*, *LZTS1*, *CER1*, *TFAP2C*) (Supplementary Fig. 1c), in agreement with RNA sequencing analyses described in detail below. Cells sorted into the S/G2/M phase and late G1 phase showed a delay in initiating differentiation. Furthermore, unsorted cells revealed a broader and lower enrichment as well as less defined expression dynamics compared to cells sorted into early G1 or the other cell cycle phases. This indicates the benefit of cell synchronisation based on the cell cycle phase, which allows examining the dynamics of transcriptional and epigenetic landscapes and enables identifying genes and genomic regions with potential stage-specific functions due to higher synchronicity of stem cell differentiation.

Next, we wondered whether the cell division was actually required for the differentiation or would the changes in culture conditions be sufficient to drive endoderm regardless of the two cell divisions. To answer these questions, we used nocodazole as utilised before^23^ for blocking cell division and analysed *MIXL1*, *T*, *EOMES*, *GSC*, *SOX17* and *FOXA2* expression by qPCR at different time points (24, 36 and 72 h) during endoderm differentiation, as well as the activating histone mark H3K4me3 and repressive histone mark H3K27me3 on their promoter region by ChIP-qPCR (Supplementary Fig. 1d). We found that blocking the cell division did not affect the induction of early markers such as *MIXL1* or *T* that are induced before any cell division, but the reduction of these early markers is less pronounced at later time points (Supplementary Fig. 1e). We observed an attenuated induction of *EOMES* and *GSC* at 36 h upon blocking the first cell division with nocodazole, and even a more pronounced difference in definitive endoderm markers *SOX17* and *FOXA2* at 72 h.

In addition, the activating histone mark H3K4me3 and repressive histone mark H3K27me3 on the promoter regions of these developmental genes reflected the transcriptional activation of these genes. The histone marks were not affected for the early genes (*MIXL1* and *T*) but having a significant reduction of H3K4me3 level and increase in H3K27me3 on *EOMES* and *GSC*, while it was more pronounced for definitive endoderm markers *SOX17* and *FOXA2* at 72 h (Supplementary Fig. 1f, g). Our results suggest that cell division impacts the efficiency and timing of definitive endoderm formation, although it does not fully abolish differentiation.

Considered together, these results confirm that our culture system can be used to differentiate cell cycle synchronised hESCs into endoderm cells, thereby providing a platform for investigating molecular regulations occurring between consecutive cell divisions.

### Genes marking differentiation are induced before cell division

Using our established culture system, we decided to investigate the transcriptional and epigenetic changes occurring during the progression of two cell divisions upon differentiation. For that, we performed genome-wide analyses, including transcriptomic characterisation by RNA-seq, chromatin accessibility changes by ATAC-seq, and epigenetic landscape dynamics by histone modification ChIP-seq (H3K4me3, H3K27me3, H3K27ac, H3K4me1 and H3K36me3) on EG1-hPSCs differentiating for 12 h (Early/Late G1 of the first cell cycle); 24 h (S/G2/M of the first cell cycle); 36 h (S/G2/M phase of the second cell cycle), 48 h (end of the second cell cycle) and 60/72 h (G1 of the third cell cycle) (Fig. 1a). Quality and reproducibility of datasets were confirmed, demonstrating the robustness and reproducibility of our approach (Methods). We first decided to characterise gene expression in hPSCs differentiating into endoderm. In agreement with previous reports, the analysis of RNA-seq data revealed precise timing and expected patterns of gene expression (Supplementary Fig. 1h, i) upon endoderm differentiation^21,24,25^. Of particular interest, *SOX2* represents the first pluripotency marker to decrease upon induction of differentiation, in agreement with its known function in repressing mesendoderm specification^26^. A decrease in *SOX2* was rapidly followed by the induction of mesendoderm markers at 12 h starting with *T* and followed by *MIXL1* and *EOMES* (Fig. 1c, d). Interestingly, mesendoderm markers (*T*, *EOMES* and *MIXL1*) start to increase before 24 h and thus before the first division. On the other hand, pluripotency markers (*OCT4/POU5F1* and *NANOG*) continue to be expressed after the first cell division (24 h) in agreement with previous reports suggesting that pluripotency factors play a role in inducing mesendoderm specification^11,27^. However, pluripotency markers strongly decreased after the first division (36 h) while endoderm markers (*GATA4/6* then *SOX17* and *FOXA2*) were induced, suggesting that endoderm specification could start before the second division. These observations confirm that the first cellular division, upon differentiation produces mesendoderm cells (*OCT4* + /*T* + /*SOX2*-), while the second division gives rise to endoderm cells (*SOX17*+/*GATA6*+/*OCT4*−). To corroborate these observations, we assigned cell-cycle scores to previously published single-cell RNA-seq data of unsynchronised induced PSCs differentiated towards endoderm, and classified each cell in either G1, S or G2/M phase^21^. In this experiment, most cells commence differentiation in the S phase (Supplementary Fig. 2a). This analysis shows that *T*, *EOMES* and *MIXL1* are highly expressed at 24 h only in S/G2/M, while *SOX17, GATA4* and *GATA6* are higher in G1 at 48 h, very likely after their second division (Supplementary Fig. 2b, c). Differential gene expression analysis in our bulk RNA-seq data showed that most variation occurs during the G1 phase in early differentiation (0–12 h) and before/after first and second divisions in most cells (24–36 h and 48–60 h, respectively) (Fig. 1e, f).

In addition, we performed differential gene expression analysis between our synchronised data at 24 h and the unsynchronised mesendoderm cells in ref. ^28^ and found 2157 differentially expressed genes. Regarding the pluripotency factors, while *OCT4*/*POU5F1* presented high expression in both conditions, *SOX2* is more expressed in our synchronised cells, while *NANOG* is more expressed in the unsynchronised condition (Supplementary Fig. 2d, e). Interestingly, mesendoderm markers were not changing significantly between the two conditions (Supplementary Fig. 2e). However, many endoderm (*SOX17*, *GATA6*, *CER1*, *FOXA2*, *CXCR4* and *GSC*) and mesoderm (*HAND1*, *RUNX1* and *TBX5*) markers were more highly expressed in the unsynchronised set up. This suggests that our synchronised experimental setup with FUCCI might be a more homogenous mesendodermal cell population, of cells awaiting the necessary differentiation cues to become either endoderm or mesoderm.

To summarise, we found that each specification phase (pluripotency to mesendoderm, and mesendoderm to endoderm) starts before the cellular division, which ultimately produces a new cell type.

### Synchronisation identifies new regulators of endoderm

To further mine our RNA-seq data and identify new factors possibly involved in endoderm differentiation, we performed *k*-means clustering of 6317 differentially expressed genes (0, 12, 24, 36, 48, 60 and 72 h). This analysis revealed 13 gene clusters, with several gene clusters displaying dynamic and transitory expression (Fig. 1h and Supplementary Fig. 1h–j). *NOTUM*, *WLS*, *BAMBI*, *CRABP1/2*, *DACT1*, *NKD* and *ID1/2/3/4* were immediately and transitorily expressed upon induction of differentiation (clusters 5, 7; Fig. 1h; Supplementary Fig. 1j and Supplementary Data 1). Except for *ID1*, the expression of these genes has not been described previously in the context of endoderm differentiation of unsynchronised hPSCs^21,24^. Their rapid decrease after induction might have obscured their expression in previous studies, despite they could be involved specifically in the earliest step of differentiation. We also observed gene clusters with expression patterns similar to those of known master regulators of endoderm specification, such as *SOX17*, and thus hypothesised that such genes could have an important role. As a representative of a potential regulator of endoderm differentiation, we characterised *LZTS1* in more detail. *LZTS1* appeared to be particularly interesting since it is expressed in the mouse primitive streak, it is bound by EOMES^11^, and its expression is strongly induced after 36 h of differentiation, following closely *GATA4/6* and *SOX17* induction (Cluster 3; Fig. 1h and Supplementary Data 1). Rapid upregulation of *LZTS1* after 36 h of differentiation was confirmed in a previously published single-cell data set (Fig. 1g, ref. ^21^) and by western blot (Fig. 1i), while gain of function experiments in hPSCs showed that *LZTS1* overexpression induces an important change in morphology of pluripotent stem cells and an increase in endoderm markers’ expression. Furthermore, this overexpression increases endoderm marker induction during endoderm differentiation, thereby suggesting a potential function for LZTS1 in endoderm specification (Fig. 1j). LZTS1 overexpression led to an increase in EOMES, SOX17 and GATA4 proteins at 72 h time point of definitive endoderm analysed by qPCR and Western blotting (Supplementary Fig. 1k, l). We also analysed the impact of LZTS1 overexpression on pancreatic differentiation by qPCR and western blotting, and found that it increased the differentiation efficiency shown by elevated expression of PDX1, MafA and INSULIN (Supplementary Fig. 1m, n), thus indicating that LZTS1 overexpression increases beta-cell specification. Taken together, these analyses reveal unique transcriptional waves during early cell fate decisions, which include previously unknown potential regulators of endoderm differentiation.

### Chromatin restriction of alternative differentiation paths

**Fig. 2:**
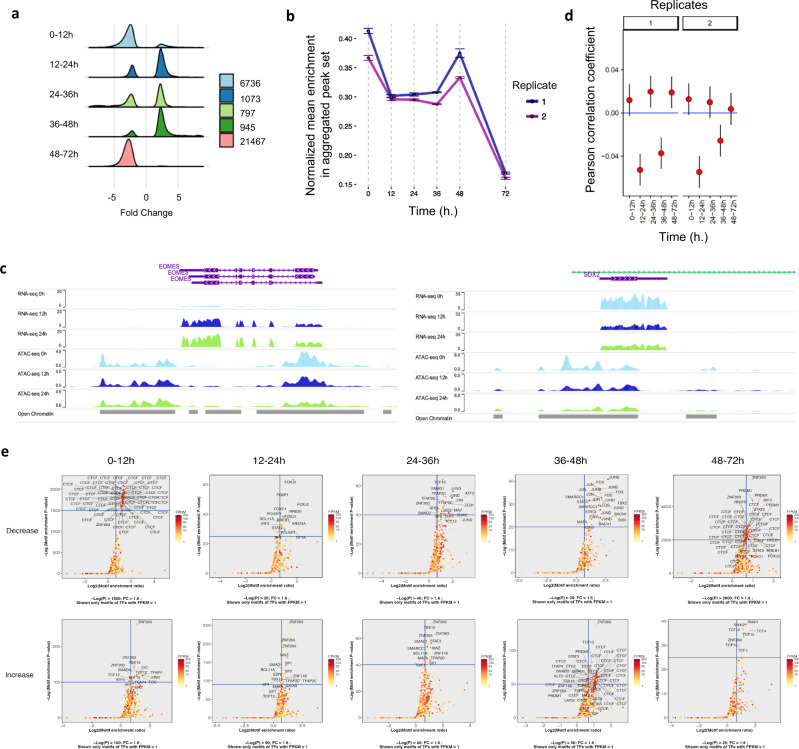
Chromatin accessibility during differentiation of G1 synchronised hPSCs. **a** The number of regions showing significant chromatin accessibility increase (“opening”; |FC| ≥2 and adj-*P* ≤ 10-4) or decrease (“closing”; |FC| ≥2 and adj-*P* ≤ 10-4) during differentiation of cell synchronised EG1-FUCCI hPSCs. **b** Normalised mean read-enrichment in ATAC-seq consensus peaks during endoderm differentiation of EG1-FUCCI hPSCs. Data are presented as mean values ± SD. **c** ATAC-seq and RNA-seq genome browser track visualisation (WashU Epigenome Browser) showing change in *EOMES* and *SOX2* (GENCODE v29) expression and associated chromatin status during the first 36 h of differentiation. The data tracks shown correspond to the first replicates. Open chromatin represents a merged list of genomic regions during differentiation. The purple bars indicate (protein) coding genes, while green represents ncRNA. **d** Pearson’s product-moment correlation between log2 fold change in RNA (reported by DESeq2) and fold change of the maximum normalised ATAC-seq signal in the two replicates in a region 10 kb upstream of the promoter of protein-coding genes. Mean and 95% confidence interval are shown. **e** Motif enrichment analyses in ATAC-seq peaks changing between different time points. The analyses shown include only motifs for DNA-binding proteins with expression above 1 FPKM. *P* values from the one-sided statistical test were calculated by HOMER using the Binomial distribution. Data in (**b**) and (**d**) depicts *n* = 3 biologically independent experiments.

Based on the dynamic expression pattern observed in our transcriptomic analyses, we investigated if the observed gene expression changes could be associated with changes in chromatin accessibility. For that, we performed ATAC-seq^29^ at the different time points of our experimental setup (Fig. 1a). Computational analyses revealed chromatin accessibility changes in 31,018 genomic regions, out of 253,349 analysed, between consecutive intervals of the time-series (|*FC*|≥2; adj-*P* ≤ 10^−4^). The majority of differential chromatin accessibility regions between time points were specific to that interval (Supplementary Fig. 4a). These changes mainly occurred in regions containing protein-coding genes and long-intergenic noncoding RNAs (Supplementary Fig. 3a). Even though the percentage of other RNAs, e.g. miRNAs, related to chromatin changes was not very high, they could still play a role in the differentiation process (Supplementary Fig. 3a). Generally, peak to nearby gene annotation in increased and invariant chromatin accessibility regions did not change with differentiation stage (Supplementary Fig. 3b). However, decreased chromatin accessibility was enriched in promoters, 5′UTRs and exons at initial and end stages of differentiation, and in intergenic regions at intermediate stages (24 and 48 h). Indistinctly of genomic annotation, chromatin mostly compacts during the first G1 phase (0–12 h) and between day 2 and day 3, while changes between day 1 and day 2 were dominated by an increase in accessibility (Fig. 2a), suggesting that different epigenetic regulations could occur between the two cell cycles leading to endoderm. Despite the increase of chromatin accessibility at intermediate stages, average chromatin accessibility was drastically reduced over the three days (Fig. 2b), confirming that hESCs display an open chromatin landscape which could provide the necessary opportunity window for differentiation induction^30^. Of note, genes close (up to 10 kb) to accessibility-decreased regions in the first G1 phase were enriched in GO terms of ‘nervous system development’, ‘system development’, ‘generation of neurons’, ‘neuron development’ and ‘neurogenesis’ (top five terms, *P* < 1.5e^−15^, hypergeometric test), suggesting that restriction of cell fate decision toward the neuroectoderm pathway through chromatin reorganisation could be one of the first events of endoderm differentiation (Supplementary Data 2). Interestingly, chromatin accessibility is also dynamically changing in undifferentiated hESCs, as shown by ATAC-seq of cells in different cell cycle phases, where the number of open regions was higher in the early cell cycle while decreasing during the S/G2/M phase, thereby confirming that chromatin could progressively close during progression towards cell division^31^.

Since our experiments suggest that the closure of chromatin accessibility at neuronal genes is one of the first events in promoting mesendoderm differentiation, we aimed to investigate the cell fate restriction in more detail. A recent study has found in mESCs that EOMES and T activation is firstly needed to then repress neuroectodermal enhancers, which are poised to be activated already in PSCs^32^. We aimed to find out whether inducible depletion of endodermal specifying factor EOMES at the second division would be able to both block endodermal differentiation and shift cells to the neural lineage, or alternatively, the closure of neuronal chromatin regions at the first cell cycle would be sufficient to impede neuronal shift even when endodermal differentiation is impaired. To investigate this, we performed a dox-inducible CRISPR/Cas9-KRAB mediated knockdown of EOMES in hESC-FUCCI cells at the second cell division during endoderm differentiation and analysed the expression of endoderm markers (*SOX17*, *FOXA2* and *GATA4*), and neuroectoderm markers (*SOX1*, *PAX6* and *SOX2*) (Supplementary Fig. 3c). These analyses indicated a strongly attenuated induction of definitive endoderm markers (*SOX17*, *FOXA2* and *GATA4*) in EOMES knockdown cells, indicating that EOMES is necessary for definitive endoderm specification (Supplementary Fig. 3d). We also investigated whether EOMES knockdown would lead the cells to undergo neuroectoderm specification by analysing neuroectoderm markers *SOX1*, *PAX6* and *SOX2* by continuous incubation of cells past Day 3 which usually signifies the stage of definitive endoderm (Supplementary Fig. 3e). We observed that while there was a moderate induction of *SOX1*, *PAX6* and *SOX2* upon EOMES knockdown after a prolonged cell incubation, the induction of neuroectoderm markers was delayed by 6 days compared to the normal duration of marker induction upon neuroectoderm induction. This 6-day delay in the upregulation of neuroectoderm markers compared to the normal progression of neuroectoderm differentiation suggests that neuroectoderm loci are reversibly closed rapidly on day 1 of endoderm differentiation, and need to be derepressed for neuroectoderm specification, which seems to require time. This derepression occurs by mechanisms that are still only partially understood, and can include the need to reverse repressive histone modifications such as H3K27me3.

Next, we correlated changes in gene expression with variant chromatin accessibility. We found that while an increase in *MIXL1*, *T* and *EOMES* expression took place in invariant chromatin regions during the first 12 h, an enhancer near *SOX2* showed significantly decreased chromatin accessibility in concert with its expression (Fig. 2c). Similarly, a change in transcription at 12–24 h for *MIXL1*, *GATA6*, *GSC*, *CER1*, *T*, *LZTS1* and *SOX17* did not display modification of chromatin accessibility (see: http://ngs.sanger.ac.uk/production/endoderm). Correlation between fold change in ATAC-seq and RNA-seq was only significant, and negative, in time intervals 12–24 h and 36–48 h (*P* < 0.005, Pearson’s product-moment correlation; Fig. 2d), which presents little transcriptional change (Fig. 1f). Similar analyses on protein-coding genes confirm the lack of correlation between change in chromatin and transcription (Supplementary Fig. 3f), thereby suggesting a weak coupling between transcriptional induction and chromatin reorganisation during early differentiation. We compared the chromatin accessibility of genomic regions between undifferentiated cells at 0 h and mesendoderm cells at 36 h of endoderm differentiation. We identified genomic regions based on ATAC-seq peaks whose accessibility did not change statistically (Supplementary Fig. 3g) and regions that were statistically changing between 0 h and 36 h, and compared the fold change in the same regions between 0 h and cells differentiated toward neuroectoderm^28^ (Supplementary Fig. 3h). By annotating the genomic regions to TSS of the nearest gene, we found endoderm genes among loci that were not significantly changing between 0 and 36 h since their chromatin was already accessible in undifferentiated hESCs. Among these genes that were accessible already in undifferentiated condition and mesendoderm stage at 36 h were mesendoderm loci *MIXL1*, *T/TBXT* and *EOMES* (Supplementary Fig. 3i) and also genomic regions near endodermal genes (e.g *GATA4*, *GATA6*, *GSC*, *SOX17* and *FOXA2*), much less accessible in neuroectoderm (Supplementary Fig. 3i). On the other hand, neuroectoderm loci such as *SOX1* (Supplementary Fig. 3j) and *SOX2* have genomic access in neuroectoderm but are closed in mesendoderm. Taken together, these results suggest that chromatin organisation changes rapidly between cell divisions during differentiation to block alternative cell fate, but not to enable the expression of genes marking mesendoderm.

### Dynamic transcription factor binding during cell divisions

To advance the understanding of the dynamic regulation necessary for endoderm differentiation, we identified overrepresented transcription factor (TF) DNA-binding motifs in dynamic chromatin accessibility regions. Motif enrichment analyses revealed that regions with increased accessibility contain motifs for effectors of signalling such as ACTIVIN/NODAL (SMAD2), BMP (SMAD1) and WNT (TCF4/12) that are known to drive endoderm differentiation (Fig. 2e). On the other hand, the CTCF motif was highly enriched in regions with decreasing chromatin accessibility during G1 phase (0–12 h, 48–72 h, Fig. 2e). Similarly, Activator protein 1 (AP-1) motifs (JUND, JUN, FOS and JUNB) were the most significantly enriched in regions displaying decreased ATAC-seq peaks associated with chromatin compaction (24–36 h and 36–48 h, Fig. 2e). Nonetheless, some TFs seem to have an opposite function at different time points during differentiation. As an example, CTCF binding motifs were also strongly associated with regions displaying increased chromatin accessibility between 36–48 h (Fig. 2e). Taken together, these motif enrichment analyses suggest that different TFs could have specific functions during the progression of endodermal differentiation.

To further identify active DNA regulatory elements, we performed digital genomic footprinting as a proxy for transcription factor binding. This approach locates depleted narrow regions within open chromatin created by TFs binding to DNA and preventing Tn5 cleavage. Genomic footprinting was first introduced in DNase-seq, and it has been performed with success in ATAC-seq^33^. Using this approach, we identified more than 2 million reproducible, non-redundant, bias-corrected, putative TF binding sites, detected by two independent software tools, and associated with genes that at the same stage were expressed at least 1 FPKM (Methods; Supplementary Fig. 4b). 75% of our 0 h/EG1 footprints co-localise with H7-hESC ENCODE footprints^34^ confirming the accuracy of our analyses (Methods). Interestingly, these analyses revealed that TF footprints were annotated more to promoters than to intronic regions in hESC (0 h) and DE (72 h), but vice versa in intermediate time points (Supplementary Fig. 5a). This suggests that “stable” cell identities have a transcriptional network relying mostly on promoters, whereas cell transitions rely more on enhancer rewiring.

**Fig. 3:**
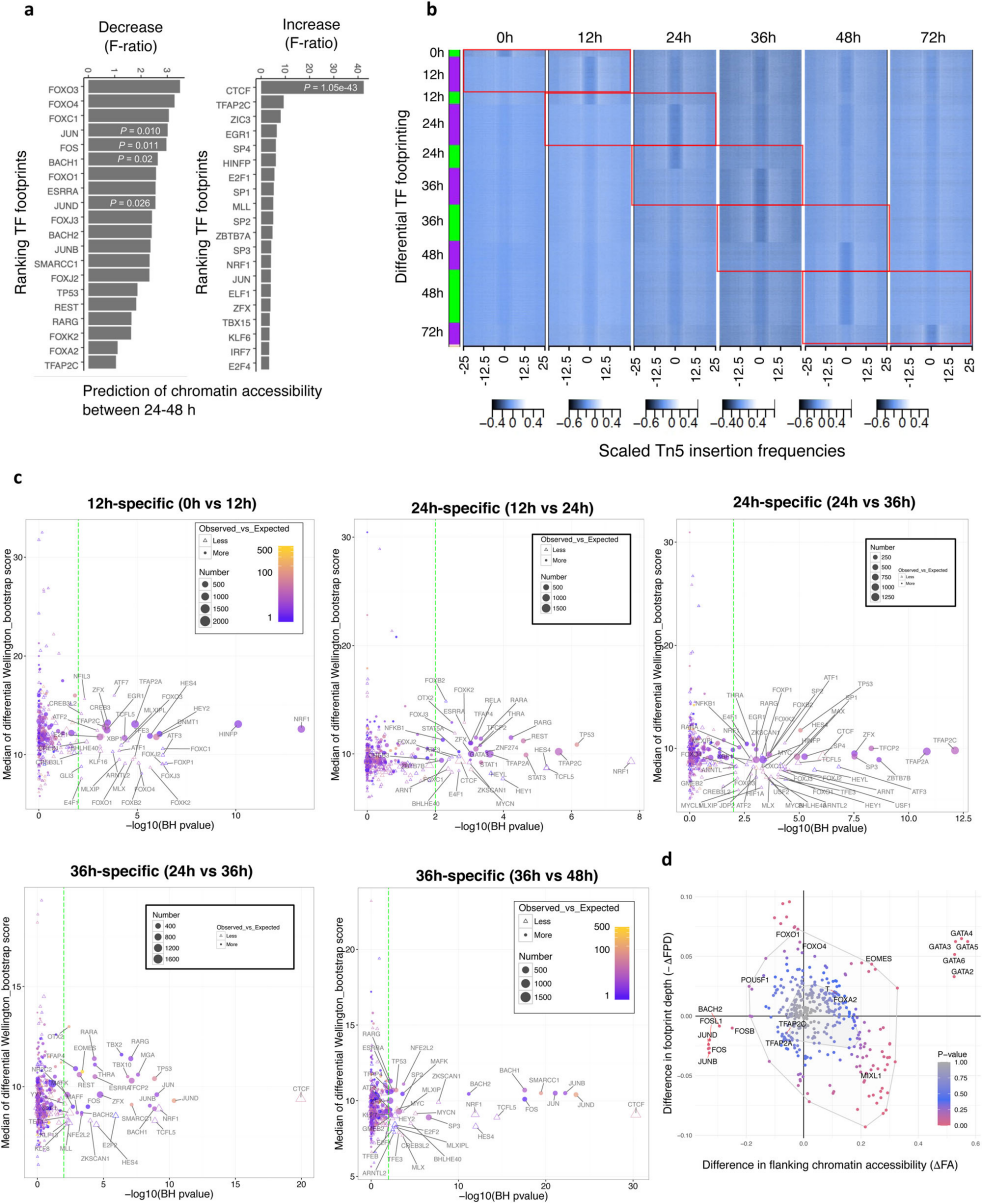
Digital genomic footprinting reveals the dynamic activity of key transcription factors during endoderm differentiation of EG1 synchronised hPSCs. **a** Top 20 (out of 353) best predictors of a functional linear model using TF footprints at 48 h to model chromatin accessibility change between 24–48 h. *P* values computed for the *F*-statistic (Wald test), to evaluate whether the fit to the data is better than what we would expect by chance, are shown for selected TFs. **b** Heatmap of normalised Tn5 insertions for differential footprints (Wellington bootstrap score *S* > 20). Red rectangles drawn indicate the results for each pairwise comparison. **c** Overrepresentation analysis for TFs associated with differential footprints shown in (**b**). Each differential footprint was first matched to the consensus list of footprints detected by FootprintMixture and Wellington (see Methods). *P* values (two-sided Chi-squared test; Benjamini–Hochberg multiple hypothesis testing correction) indicate if the proportions of footprints at each time point for a TF are significantly different when comparing differential footprints and the total number of footprints at this time point. Only TFs with adjusted *P* ≤ 0.01 have been labelled. **d** Bag plot depicting changes in flanking chromatin accessibility (*Δ*FA) and footprint depth (*Δ*FPD) in ATAC-seq of endoderm differentiation between 24 and 48 h in human motifs. Statistically significant changes in FA/FPD were evaluated by the two-sided Chi-squared test. Motifs of genes that were not differentially expressed during endoderm differentiation (RNA-seq) were removed. Outlier motif ARID5A (*Δ*FA = 0.15, *Δ*FPD = 0.27) was also removed from the plot.

We next decided to identify transcription factors whose presence results in major changes in chromatin organisation in subsequent time points during differentiation. For that, we implemented a predictive model to systematically study TF action in chromatin accessibility dynamics. A functional linear model with a scalar response^35^, also known as functional predictor regression^36^, was used to predict the log_2_|FC| of chromatin accessibility in ATAC-seq peaks between two consecutive stages for TF footprints that overlap those regions (Methods). The linear model was only considered when at least 15 footprints overlapped the regions of interest. The analysis was performed using footprints detected either at initial or final time points as a reference. We assessed the quality of the fit for each TF by computing the multiple square correlation (RSQ) and *F*-ratio (Wald test). *P* values for the *F*-statistic were calculated to evaluate whether the fit to the data is better than what we would expect by chance. We observed low RSQ, suggesting that DNA-binding of each TF in isolation does not predict well ATAC-seq change, confirming that combinatorial binding of several factors is generally necessary for the context of chromatin^37^. Nonetheless, we obtained statistically significant results for footprints of diverse TFs, including, for example, FOXA2, SMARCC1, CTCF and TP53 at 36 h, which were the best predictors for the decrease in chromatin accessibility occurring at 48 h (for details, see Methods). Footprints for proteins of the AP-1 complex found at 48 h were top predictors of chromatin compaction between 24–48 h (Fig. 3a), in agreement with our DNA motif enrichment analyses (Fig. 2e). To investigate these observations further and reveal TF dynamics genome-wide in a statistically robust manner, differential ATAC-seq footprinting was performed with Wellington_bootstrap (Fig. 3b), which is able to account for the different sequencing depths of the datasets^38^. We found that most significantly overrepresented TFs were NRF1 (12 h), TP53 and TFAP2A/C (24 h) and JUND/JUN/JUNB (36 h), members of the AP-1 transcription factor complex (Fig. 3c). Reanalysis of the data with a recent algorithm for bivariate genomic footprinting showed similar results and uncovered the GATA motif in increased chromatin accessibility at 48 h (Fig. 3d). Immunofluorescence analysis of synchronised cells revealed expression of NRF1 specially during the first 24 h, while expression of TFAP2C at the protein level was limited and null after 36 h (Supplementary Fig. 4c). These results confirm that different combinations of transcription factors could organise chromatin at specific time points upon early differentiation of hESCs. They also reveal significant effects of TF binding in chromatin modulation and suggest that AP-1 factors could have a key function in modulating chromatin accessibility after the first cell division.

### MEK/ERK/p38/AP-1 control endoderm via FOSL2/JUN and SMAD2/3

**Fig. 4:**
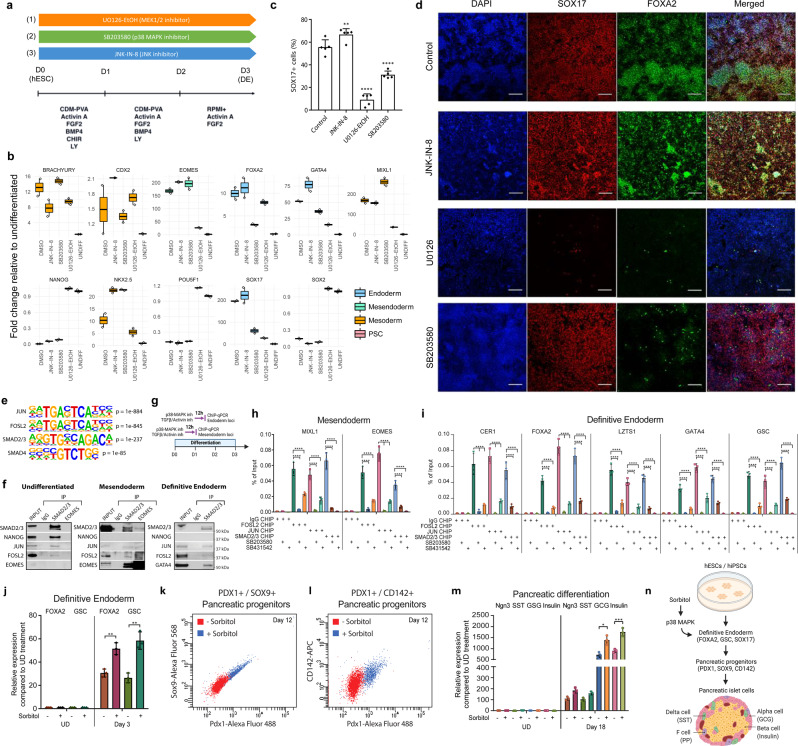
Inhibition of AP-1 complex blocks endoderm differentiation of human pluripotent stem cells via locus-specific transcriptional complexes. **a** Schematic representation of the experimental plan to characterise the functional relevance of MEK1/2, JNK and p38 pathways in endoderm differentiation. hPSCs were grown for 3 days in culture conditions inducing endoderm differentiation in the presence of small-molecule inhibitors. **b** QPCR, **c** FACS and **d** immunostaining were performed after 3 days for pluripotency markers (POU5F1/OCT4, NANOG and SOX2), mesendoderm/mesoderm markers (BRACHYURY/T, NKX2.5, CDX2 and MIXL1) and endoderm markers (SOX17, EOMES, GATA4 and FOXA2). For QPCR, the boxes show the interquartile range, with the median marked as a heavy horizontal band. Whiskers represent the highest (lowest) datapoint within 1.5 times the interquartile range of the 75th (25th) percentile. The diamonds represent each datapoint. For FACS, one-way ANOVA was performed, followed by Dunnett’s multiple comparisons test where each of the three treatment conditions was compared against the control in *n* = 5 experiments. The statistical significance for FACS marks adjusted *P* values: ** (0.0096), **** (<0.0001). For immunostaining, the scale bar represents 200 µm. Experiments represent three replicates. Statistical analysis was performed by two-way ANOVA with multiple comparisons and **** marks adjusted *P* value <0.0001. **e** Consensus binding motifs for AP-1 transcription factors (JUN and FOSL2), SMAD2/3 and SMAD4 were obtained from ATAC-seq analyses at 36 h time point with corresponding *P* values. **f** The switching of transcription factor complexes during hPSC differentiation to definitive endoderm. SMAD2/3 was immunoprecipitated from nuclear extracts of undifferentiated hPSCs, at 36 and at 72 h after initiating endoderm differentiation and analysed for the co-immunoprecipitation of NANOG, EOMES, GATA4, FOSL2 and JUN. **g** Schematic representation of the experimental outline for analysing the impact of p38-MAPK and TGFβ/Activin A signalling on SMAD2/3 and FOSL2/JUN binding and H3K27ac enrichment on mesendoderm at 24 h, and endoderm or mesoderm loci at 36 h by ChIP-qPCR. Cells were treated with p38-MAPK and TGFβ/Activin A inhibitors for 12 h in the presence of differentiation signals before sample collection. **h** FOSL2/JUN and SMAD2/3 cooperative binding to *MIXL1* and *EOMES* regulatory regions at mesendoderm differentiation stage during hPSC differentiation. Early G1 phase synchronised cells were differentiated to endoderm for 12 h to receive the pluripotency exit signalling, and then treated with p38-MAPK inhibitor SB203580 or TGFβ/Activin signalling inhibitor SB431542 for 12 h, followed by cell fixation for ChIP-qPCR (before first cell division). Analyses reveal that p38-MAPK and TGFβ/Activin signalling regulate the cooperative binding of AP-1 TFs FOSL2/JUN and SMAD2/3 to mesendoderm genes at 24 h time point; *n* = 3 biologically independent experiments. **i** FOSL2/JUN and SMAD2/3 cooperative binding to *CER1*, *FOXA2*, *LZTS1*, *GATA4* and *GSC* regulatory regions at endoderm differentiation stage during hPSC differentiation. Early G1 phase synchronised cells were differentiated to endoderm for 24 h, and then treated with p38-MAPK inhibitor SB203580 or TGFβ/Activin signalling inhibitor for 12 h, followed by cell fixation for ChIP-qPCR (before second cell division). p38-MAPK and TGFβ/Activin signalling regulate the cooperative binding of AP-1 TFs FOSL2/JUN and SMAD2/3 to definitive endoderm genes at 36 h time point. *n* = 3 biologically independent experiments. **j**–**m** Activation of p38-MAPK signalling improves definitive endoderm and pancreatic differentiation. Cells were treated with 200 mM Sorbitol during 24 to 72 h of endoderm differentiation and analysed by **j** qPCR of *FOXA2* and *GSC* expression at day 3, **k**, **l** FACS of PDX1, SOX9 and CD142 co-expression at day 12, **m** qPCR of *NGN3*, *SST*, *GSG* and Insulin expression at day 18 of pancreatic differentiation. Experiments represent three replicates. **n** Activation of p38-MAPK signalling by Sorbitol improves definitive endoderm differentiation and the formation of endoderm-derived pancreatic cell types. Schematic representation of hESC and hiPSC differentiation to definitive endoderm, pancreatic progenitors and pancreatic islets of the Langerhans that contain ɑ, β, δ and F cells expressing their corresponding secreted factors such as insulin by β cells. Activation of p38-MAPK by Sorbitol improved definitive endoderm differentiation and the formation of subsequent pancreatic cells. Adapted from 'Pancreatic Islet of Langerhans', by BioRender.com (2023). Retrieved from https://app.biorender.com/biorender-templates. Statistical analyses in (**h**, **i**, **j**, **m**) were performed by two-way ANOVA with Tukey’s multiple comparisons tests and * marks adjusted *P* value <0.05 and ** marks adjusted *P* value <0.01, *** marks adjusted *P* value <0.001, **** marks adjusted *P* value <0.0001. Data were presented as mean values ± SD. Source data are provided as a Source Data file.

To corroborate the functional relevance of computational ATAC-seq analyses, we investigated the importance of AP-1 signalling pathways in DE differentiation. For that, two separate hPSC lines were differentiated in the presence of inhibitors for three different pathways which are known to control the AP-1 complex: MEK1/2 (U0126-EtOH), p38-MAPK (SB203580) and JNK (JNK-IN-8) (Fig. 4a). These analyses in both hPSC lines at 72 h time point revealed that p38-MAPK inhibition blocked the expression of DE markers (*GATA4*, *SOX17*, *CDX2* and *FOXA2*, Fig. 4b–d and Supplementary Fig. 5b, c) without affecting the increase of primitive streak markers (*T*, Fig. 4b and Supplementary Fig. 5b) or the decrease of pluripotency markers (*OCT4*/*POU5F1*, *NANOG*, *SOX2*, Fig. 4b and Supplementary Fig. 5b) while promoting mesoderm markers’ expression (*MIXL1*, *NKX2.5*, Fig. 4b and Supplementary Fig. 5b). On the other hand, JNK inhibition marginally increased the expression of endoderm markers, and inhibition of MEK1/2 blocked DE differentiation while limiting pluripotency markers’ decrease (Fig. 4b–d and Supplementary Figs. 5b, c,  7b). We also examined the effects of SB203580, SB431542, JNK-IN-8 and UO126-EtOH on gene expression at a different time point, at 24 h of endoderm differentiation, after treating the samples for 12 h. The results indicated that the expression of *T* is not significantly affected, while *MIXL1* and *EOMES* were reduced by SB203580 and strongly reduced by U0126-EtOH (Supplementary Fig. 5d). We investigated the effects on pluripotency factors at 24 h of endoderm differentiation and found that U0126-EtOH blocked the decrease of *SOX2*, which is the earliest of the pluripotency factors to decrease expression after endoderm differentiation (Supplementary Fig. 5e). Flow cytometry experiments at 24 h of endoderm differentiation indicated that U0126-EtOH blocked EOMES induction whereas JNK-IN-8 led to a modest increase in EOMES induction (Supplementary Figs. 5f,  7c).

These data suggest that the p38-MAPK-AP1 pathway is necessary for endoderm specification. Combined, these experiments and the computational analyses shed light on the different effects exerted by the signalling controlling the AP-1 complex. MAPK-ERK seems necessary to exit pluripotency during endoderm specification^39,40^ while MAPK-p38 promotes endoderm induction by inhibiting mesoderm. This diversity of functions could be achieved by dynamic interactions between the members of the AP-1 complex at different time points during the first two cell divisions upon differentiation. To investigate the possibility that transcription factors might bind to target loci dynamically at different time points during endoderm differentiation, we identified enrichment analysis of known motifs with HOMER based on Wellington footprints for each time point during differentiation. This uncovered highly statistically significant footprints of SMAD2/3, the central regulators of definitive endoderm formation via TGFβ/Activin A signalling, as well as AP-1 transcription factors JUND/JUN/JUNB and FOSL2/FOSL1/FOS as shown for 36 h time point (Fig. 4e). We observed the footprints for SMAD2/3, FOSL2 and JUN also nearby key mesendoderm/primitive streak loci (*MIXL1* and *EOMES*), mesoderm loci (*MESP1* and *MESP2*) and definitive endoderm loci (*CER1*, *FOXA2*, *LZTS1*, *GATA4* and *GSC*). We investigated the expression of endoderm markers (Supplementary Fig. 5g) and mesoderm markers (Supplementary Fig. 5h) at 36 h of endoderm differentiation upon the treatment with p38-MAPK inhibition SB203580 and TGFβ/Activin A-SMAD2/3 pathway inhibitor SB431542. As expected, the TGFβ/Activin A-SMAD2/3 pathway inhibitor SB431542 abolished the induction of definitive endoderm markers *CER1*, *FOXA2*, *LZTS1*, *GATA4* and *GSC*, while p38-MAPK inhibition with SB203580 also attenuated the induction of these genes compared to control endoderm differentiation treated with DMSO (Supplementary Fig. 5g). In contrast, SB203580 and SB431542 treatment increased mesoderm markers *MESP1* and *MESP2* (Supplementary Fig. 5h). Flow cytometry analyses indicated that p38-MAPK inhibition with SB203580 led to reduced levels of GATA4 whereas SB431542 and U0126-EtOH blocked GATA4 expression (Supplementary Fig. 5i). These data suggest that the p38-MAPK-AP1 and TGFβ/Activin A-SMAD2/3 pathway induce endoderm over mesoderm specification and for full dismantling of the pluripotency network. As the induction of mesendoderm/primitive streak loci (*MIXL1* and *EOMES*), mesoderm loci (*MESP1* and *MESP2*) and definitive endoderm loci (*CER1*, *FOXA2*, *LZTS1*, *GATA4* and *GSC*) occur at different stages of definitive endoderm specification, we hypothesised that AP-1 factors form complexes with SMAD2/3 and other developmental transcription factors during differentiation. This could potentially help to explain AP-1 targeting to developmental loci during endoderm differentiation. Co-immunoprecipitation experiments of SMAD2/3 revealed the switching of transcription factor complexes during hPSC differentiation to definitive endoderm (Fig. 4f). SMAD2/3 substitutes its interaction partners from the pluripotency factor NANOG in undifferentiated hPSCs to EOMES at mesendoderm stage, and to GATA4 at definitive endoderm stage. FOSL2 and JUN also bind to these transcription factors at the corresponding stages (Fig. 4f), suggesting that the targeting of this transcription factor complex is changing during stepwise cell specification through multiple cell divisions depending on the expression of developmental transcription factors and activation of signalling pathways. FOSL2/JUN bind to SMAD2/3, but since SMAD2/3 changes partners during the endoderm differentiation process from NANOG in undifferentiated hPSCs to EOMES in mesendoderm stage and further to factors such as GATA4 in definitive endoderm, this is likely to regulate the genomic regions where these transcription factors complexes bind. It means that FOSL2/JUN could ‘benefit’ from this exchange of SMAD2/3 cofactors since that helps to target these AP-1 TFs dynamically to different developmental loci during endoderm specification.

Since motif analysis indicated the presence of FOSL2, JUN and SMAD2/3 binding sites at regulatory sequences near primitive streak/mesendoderm loci, we designed primers to amplify the regions expected to bind to regulatory regions (Supplementary Information—Supplementary Table 3). We performed ChIP-qPCR on synchronised cells at 24 or 36 h after initiating endoderm differentiation, and the addition of p38-MAPK inhibitor SB203580 or TGFβ/Activin signalling inhibitor SB431542 for the last 12 h before cell collection (Fig. 4g). ChIP-qPCR on synchronised cells at 24 h after initiating endoderm differentiation corresponds to cells that have not yet undergone cell division according to cell tracer signal. JUN ChIP, FOSL2 ChIP and SMAD2/3 ChIP revealed the binding of these transcription factors to *MIXL1* and *EOMES* regulatory regions, whereas p38-MAPK inhibition with SB203580 abolished the binding of JUN and FOSL2 to these loci (Fig. 4h). Interestingly, p38-MAPK inhibition also reduced SMAD2/3 binding to *MIXL1* and *EOMES*, indicating that this signalling pathway and/or JUN-FOSL2 complex helps to recruit or stabilise SMAD2/3 binding. In turn, TGFβ/Activin signalling inhibitor SB431542 treatment not only caused the loss of SMAD2/3 binding but also reduced JUN and FOSL2 binding, suggesting a cooperative binding of the SMAD2/3-FOSL2-JUN complex. We also found SMAD2/3 and AP-1 binding motifs at mesoderm loci and definitive endoderm loci. ChIP-qPCR at 36 h of differentiation which corresponds to cells that have undergone one cell division by cell tracer signal, indicated the binding of SMAD2/3 as well as FOSL2 and JUN to definitive endoderm loci *CER1*, *FOXA2*, *LZTS1*, *GATA4* and *GSC* (Fig. 4i) and mesoderm loci *MESP1* and *MESP2* (Supplementary Fig. 6a). p38-MAPK inhibition with SB203580 resulted not only in the reduced binding of JUN and FOSL2 to mesoderm and definitive endoderm loci, but also attenuated binding of SMAD2/3 to the same loci, while TGFβ/Activin signalling inhibitor SB431542 treatment reduced SMAD2/3 binding but also FOSL2 and JUN binding (Fig. 4i and Supplementary Fig. 6a). Hence, TGFβ/Activin-SMAD2/3 and p38-MAPK-FOSL2/JUN cooperate at multiple stages of definitive endoderm specification.

Next, we looked at the impact of p38-MAPK inhibition and TGFβ/Activin inhibition on the activating histone mark H3K27ac by ChIP-qPCR. By using the same timing of treatments as for SMAD2/3 and FOSL2/JUN ChIP experiments, we found that the H3K27ac levels were decreased on primitive streak/mesendoderm loci (*MIXL1* and *EOMES*) as well as definitive endoderm loci (*CER1*, *FOXA2*, *LZTS1*, *GATA4* and *GSC*) but they were increased on mesoderm loci *MESP1* and *MESP2* (Supplementary Fig. 6b–d). To test the involvement of the AP-1 pathway in mesoderm regulation, we treated undifferentiated cells cultured in embryoid body media with AP-1 activators 12-*O*-tetradecanoylphorbol-13-acetate (TPA) and PDGF followed by qPCR analyses (Supplementary Fig. 6e). AP-1 activators TPA and PDGF led to a relative reduction in mesoderm marker expression (*MESP1* and *MESP2*) suggesting that the AP-1 transcription factor pathway reduces mesoderm specification and favours specification to definitive endoderm.

Collectively, these data indicate temporally dynamic and locus-specific binding of TFs during pluripotent stem cell differentiation to mesendoderm and further cell fate specification to endoderm over mesoderm (Supplementary Fig. 6f).

### p38-MAPK optimises pancreatic beta-cell differentiation

One of the long-term goals in biomedicine is using hPSCs for differentiating them into functional cell types such as pancreatic beta-cells for biomedical applications, but the low efficiency of cell differentiation has remained a challenge. To examine the practical utility of our findings, we differentiated hPSCs to endoderm and further to pancreatic lineage as described previously^18^, while also adding 200 mM sorbitol to the differentiation media during days 2–3 to induce the activity of p38-MAPK signalling. The analysis indicated an increase in definitive endoderm markers *FOXA2* and *GSC* compared to the control differentiation at day 3 (Fig. 4j), increased expression of central transcription factors of pancreatic tissue formation such as SOX9 and PDX1 at day 12 of differentiation (Fig. 4k, l and Supplementary Fig. 7d) and pancreatic maturation markers *NGN3*, *SST* and *GCG* and Insulin at day 18 of pancreatic differentiation (Fig. 4m). Differentiating patient-derived iPSCs to functional cell types such as insulin-producing beta-cells for restoring the function of the pancreatic endocrine cells in Type I diabetes patients is an attractive long-term aim in the regenerative medicine field. Hence, our findings have practical utility for more efficient generation of hIPSC-derived cell types such as pancreatic insulin-producing beta-cells for biomedical applications (Fig. 4n).

### Consecutive cell divisions and dynamic histone modifications

**Fig. 5:**
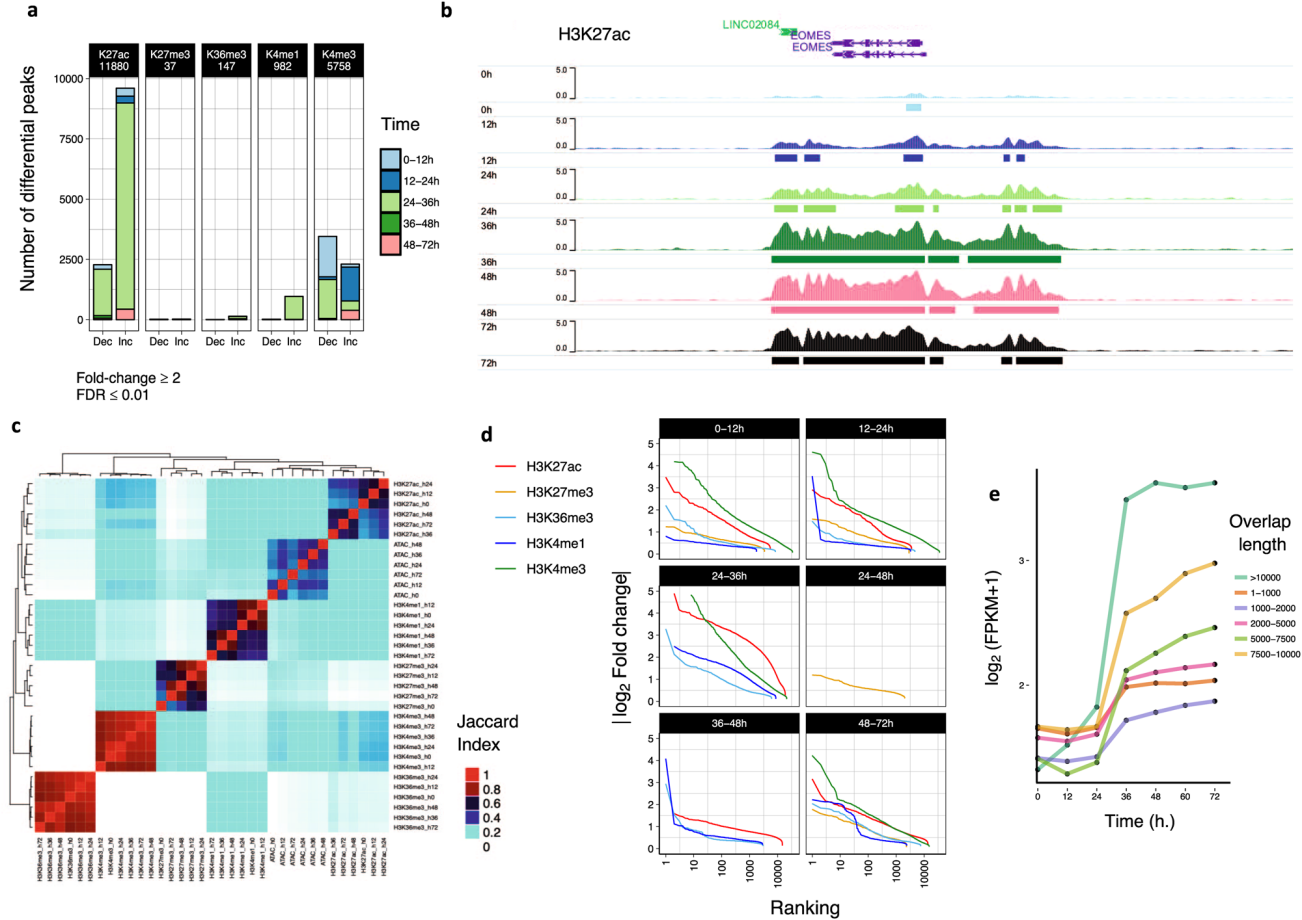
Histone modification dynamics during differentiation of EG1 synchronised hPSCs. **a** The number of genomic regions containing dynamic chromatin marks in consecutive time points based on histone mark ChIP-seq. **b** H3K27ac ChIP-seq for *EOMES* locus (GENCODE v29; first replicate shown). **c** Hierarchical clustering of Jaccard Index values obtained for overlaps between ChIP-seq and ATAC-seq regions. **d** The absolute value of log_2_ fold-enrichment ratios for significant sites (FDR ≤0.01) obtained after differential histone modification analysis. **e** Gene expression versus length of the direct overlap between H3K4me1 and H3K27ac both increase between 24 and 36 h (for protein-coding genes, 10 kb around).

To investigate potential changes in the epigenetic landscape occurring during differentiation, we performed ChIP-seq experiments for five histone modifications (H3K4me3 for active promoter regions, H3K4me1 and H3K27ac for enhancers, H3K27me3 for repressed regions, and H3K36me3 for gene bodies of actively transcribed genes). We identified differential ChIP-seq regions between consecutive stages (|FC|≥2; FDR ≤0.01, *G*-tests, Benjamini–Hochberg multiple testing correction) and observed that the transition from pluripotency to DE involved several waves of histone modification changes (Fig. 5a). H3K4me3 and H3K27ac marks were the most dynamic during differentiation. H3K4me3 decreases during the initiation of differentiation (0–12 h), suggesting that the first step of the differentiation consists of inhibiting alternative fate choice, in agreement with our ATAC-seq analyses. In addition, early developmental loci rapidly acquire H3K4me3 and H3K27ac marks during 12–24 h, confirming that epigenetic modifications characterising differentiation could start before the first division. Accordingly, regions surrounding key genes such as *T*, *GSC*, *SOX17*, *EOMES*, *MIXL1*, *GATA6*, *GATA4*, *LHX1* and *FOXA2* acquire H3K27ac rapidly upon differentiation (0–12 h, Fig. 5b; see http://ngs.sanger.ac.uk/production/endoderm). Major acquisition/increase of enhancer marks (H3K27ac and H3K4me1) also occurs after the first division and before the second division (24–36 h), and approximately before the third cell division (48–72 h) (Fig. 5a). Clustering of Jaccard indexes using all the reproducible peaks in ChIP-seq and ATAC-seq (those with >100 regions) to quantify the overlap of chromatin marks during differentiation confirmed that enhancer marks (H3K27ac, H3K4me1) and open chromatin are very dynamic (Fig. 5c). In addition, despite the lack of quantitative peak changes, we could observe that H3K27me3 chromatin regions at 0 and 72 h were dissimilar, and that H3K36me3 peak sets clustered into two groups: before and after the first cell division (Fig. 5c). Nevertheless, we found reproducible and statistically significant peak changes for these marks, although they presented low fold-enrichment ratios (Fig. 5d). Simultaneous changes in H3K27ac/H3K4me3 and H3K27ac/H3K4me1 seem to associate more than with any other marks, and changes in chromatin accessibility were also linked with the deposition of these marks. As expected, H3K27me3 and H3K36me3 or H3K27me3 and H3K27ac were mutually exclusive.

We then decided to correlate the change in gene expression with histone mark acquisition focusing on H3K4me1 and H3K27ac since 784 out of 954 H3K4me1 increased regions were coincident with H3K27ac increase between 24–36 h. We observed that, when both marks increased simultaneously, genes in close proximity increased its expression proportionally to the size of the overlap between the two chromatin marks (Fig. 5e). Interestingly, this correlation starts immediately upon differentiation suggesting that tissue super-enhancers (SE, marked by H3K27ac and H3K4me1 spanning an intersection of >3 kb)^41,42^ seem to be initiated before division to become fully established only after the first division, thereby following the induction of expression for key developmental regulators (Fig. 1c). Taken together, these data suggest the existence of a hierarchy between histone marks during differentiation. H3K27ac appears on key genes marking nascent mesendoderm before or during the induction of their expression immediately after the first G1 phase. On the other hand, H3K27me3 and H3K36me3 present less acute changes and seem to fall behind gene expression and/or chromatin organisation.

### Transient super-enhancers after divisions and cell identity

**Fig. 6:**
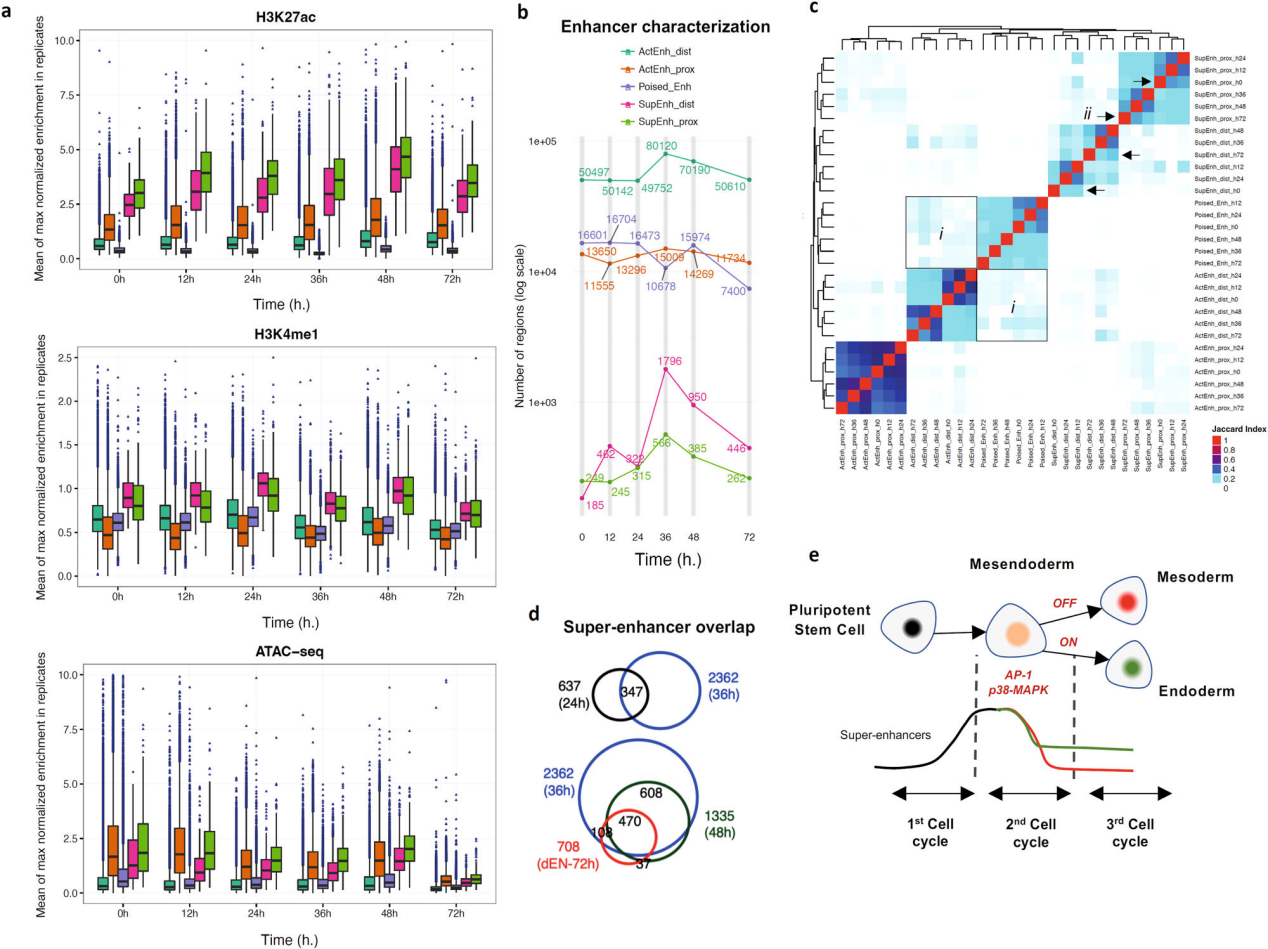
Epigenome dynamics upon differentiation reveal super-enhancers assembly and loss to establish a new cellular identity. **a** Distribution of H3K27ac, H3K4me1 and ATAC-seq signal in the enhancer regions shown in (**b**). The boxes show the interquartile range, with the median marked as a heavy horizontal band. Whiskers represent the highest (lowest) datapoint within 1.5 times the interquartile range of the 75th (25th) percentile. Statistical outliers are plotted separately as individual points beyond the whiskers on the boxplot. *n* = 2 biologically independent samples. **b** Enhancer classification during endoderm differentiation: ActEnh_dist (H3K27ac+, H3K4me1+, H3K4me3−, <3 kb); ActEnh_prox (H3K27ac+, H3K4me1+, H3K4me3+, <3 kb); Poised_Enh (H3K27ac−, H3K4me1+); SupEnh_dist (H3K27ac+, H3K4me1+, H3K4me3−, > 3 kb); SupEnh_prox (H3K27ac+, H3K4me1+, H3K4me3+, > 3 kb). **c** Hierarchical clustering of Jaccard Index values obtained for overlaps between different enhancer regions. The squares (i) highlight the conversion of poised into active enhancers, while the arrows (ii) indicate the similarity of super-enhancers at 0 and 72 h with other time points. **d** Endoderm-specific super-enhancers are a subset of those established at 36 h. **e** Schematic model of the super-enhancer establishment during endoderm differentiation at each consecutive cell division.

Next, we aimed to use our ChIP-seq data to characterise the enhancer regulatory landscape defining cellular identity acquisition. For that, we defined at each time point poised enhancers (PEs, H3K27ac− and H3K4me1+), active enhancers (AEs, H3K27ac+, H3K4me1+ <3 kb), and super-enhancers (SEs, H3K27ac+, H3K4me1+ >3 kb). As H3K4me3 has been detected in promoters^43^, we subdivided AEs and SEs as proximal or distal according to the presence of H3K4me3 (Fig. 6a). Distal AEs and distal SEs were the most dynamic regulatory regions with their number increasing immediately after differentiation to peak at 36 h after one cell division (Fig. 6b), and then decreasing at 72 h. Proximal AEs and poised enhancers were relatively stable during the time course. These results confirm that SE establishment starts immediately upon differentiation to be consolidated after the first division to establish a new cellular identity. We then performed clustering of Jaccard indexes to identify similarities between regulatory regions. Using this approach, we observed that poised enhancers partially converted to distal AEs after one division, especially at 36 h (Fig. 6c, i). Both distal and proximal SEs at 0 and 72 h were less similar to those at 12/24 h and 36/48 h, suggesting that acquisition and partial loss of SEs is occurring actively at each cell division resulting in a new cellular identity (Fig. 6c, ii). Nonetheless, while SEs were different before and after first cell division, SEs at 48 h were a subset of those established at 36 h (Fig. 6d). For example, key mesendoderm/endoderm loci such as *CER1*, *EOMES*, *LZTS1* and *MIXL1* contain several SEs established and maintained after first cell division (FC >4; *P* ≅ 0) (http://ngs.sanger.ac.uk/production/endoderm) (Supplementary Fig. 6g). Other SEs at 36 h include *EOMES*, *LHX1*, *GSC*, *ZIC3*, *OTX2*, *DKK1*, *WLS*, *MYC*, *HAND1*, *WNT3*, as well as some members of the MAPK family, among others. When we intersected the transcription factors footprints with the enhancers, we observed enrichment in distal super-enhancers for CTCF, TFAP2A, TFAP2C, JUN, TP53, ZFX, TFAP4, RARG, FOSL1/2, JUND, BACH1, ZIC3, MAFK, FOS, JUNB, ESRRA and SMARCC1 as soon as 12 h (Supplementary Fig. 6h), and CTCF, TFAP2C, REST, TFAP4, RARA, RARG, RFX5, MGA and TCP2 at 36 h (Supplementary Fig. 6i, j). Thus, SEs seem to be established following two different regulatory mechanisms between the first and second division (Fig. 6e). A large number of new SEs are established after the first division at the transitory mesendoderm stage, while SEs characterising endoderm cells are simply maintained during the second division when other mesendoderm specific enhancers are progressively lost. Thus, cellular identity acquisition could be achieved by the creation or selection of super-enhancers starting before division, thereby suggesting the existence of different epigenetic regulations between successive cell cycles upon differentiation.

## Discussion

**Fig. 7:**
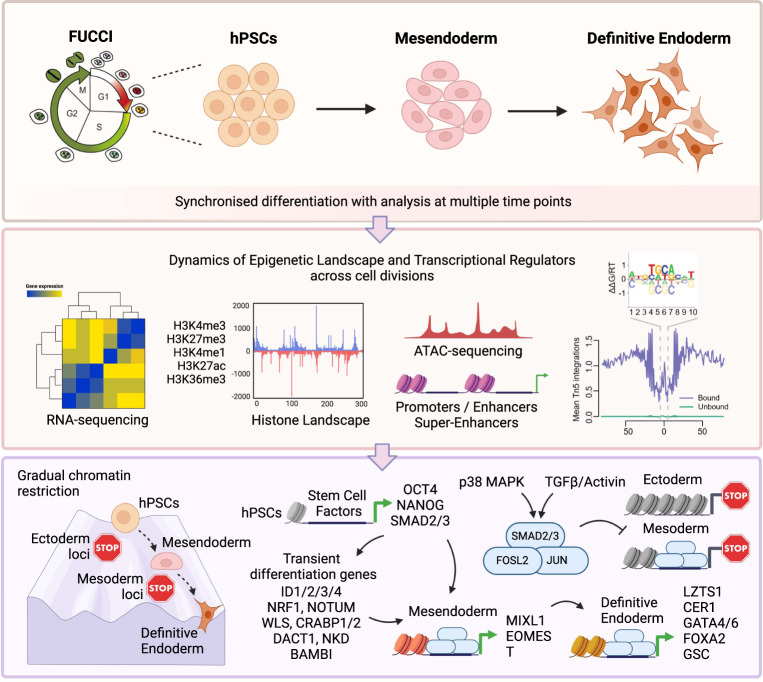
Schematic depiction of the study design, analyses methods and results from synchronised hPSC differentiation to definitive endoderm. We took advantage of the Fluorescent Ubiquitination-based Cell Cycle Indicator (FUCCI) reporter to develop a culture system allowing the differentiation of human embryonic stem cells (hESCs) synchronised in their early G1 phase of the cell cycle to assess the epigenome and transcriptome dynamics during the first two divisions leading to definitive endoderm. Our data comprise multiple time points spanning each successive cell cycle and include simultaneous analysis of RNA-seq, ATAC-seq and ChIP-seq. A summary of the results is shown together with a map of Waddington’s epigenetic landscape for endoderm cell fate specification upon differentiation of human pluripotent stem cells. Overall, these data reveal key successive interplays between epigenetic modifications during differentiation and provide a valuable resource to investigate mechanisms in germ layer specification. Created with BioRender.com.

Our data provide a comprehensive epigenetic resource outlining the progressive acquisition of endoderm identity upon consecutive cell divisions (Fig. 7). Our analyses also show that early differentiation to definitive endoderm follows gradual and stepwise transitions during two complete cell cycles. Differentiation is initiated in the G1 phase when the expression of mesendoderm markers is initiated at 12 h, long before the first cell division occurs. This timing implies that the first step of mesendoderm specification could be achieved through the reorganisation of the pluripotency network induced by changing culture conditions. Nonetheless, we did observe the transitory induction of early genes upon induction of differentiation. Synchronisation allowed us to uncover new pathways and regulators of differentiation that would have otherwise not been found. RNA-seq of synchronously differentiating hESC-FUCCI cells uncovered several gene clusters displaying a dynamic and transitory expression. *NOTUM*, *WLS*, *BAMBI*, *CRABP1/2*, *DACT1*, *NKD* and *ID1/2/3/4* were immediately and transitorily expressed upon induction of differentiation. Except for *ID1*, the expression of these genes has not been described previously in the context of endoderm differentiation of unsynchronised hESCs. Most of these genes are known to control signalling pathways, such as WNT for *NOTUM* or BMP for *BAMBI*^44–46^. In addition, some of these genes (*ID1*/*3*/*4*) are controlled by BMP signalling, which is added to our culture condition. Thus, these genes might mark merely a response to the change in culture conditions rather than actively direct the differentiation process^47^. Alternatively, they could also repress neuroectoderm differentiation. Accordingly, we found that initiation of differentiation is associated with a decrease in chromatin accessibility around neuronal-related genes, suggesting that one of the first differentiation events is blocking alternative cell fate specifications. Moreover, the rapid decrease in *SOX2* expression is likely to play a key function in this process. *SOX2* is a key regulator of neuroectoderm specification while also being a key inhibitor of mesendoderm. On the other hand, we could not detect major changes in chromatin organisation associated with the induction of mesendoderm markers suggesting that the corresponding genomic regions are already accessible in pluripotent stem cells. This invariant chromatin accessibility could imply that the reorganisation of the pluripotency network merely needs to activate the transcription of mesendoderm genes without major changes in chromatin accessibility. This mechanism will enable fast and rapid induction of differentiation following a change in culture conditions. Such a process is likely to be essential in a constantly changing environment such as the gastrulating embryo. On the other hand, since chromatin opening is not key for the activation of differentiation genes, it raises the question of what is then blocking differentiation early on. It is likely that several mechanisms block differentiation, which includes the lack of sufficiently high expression of lineage-specific TFs that would initiate differentiation, and also the presence of the core pluripotency network even upon low and variable expression of lineage-specific TFs. Since the ESC genome is transcriptionally globally hyperactive with widespread transcription in coding and noncoding regions in ESCs, there could also be post-transcriptional mechanisms that impact alternative splicing, protein translation and post-translational mechanisms through pluripotency-inducing signalling pathways that all contribute to blocking differentiation while maintaining the pluripotent state.

In general, there is no change in the decrease in the accessibility of miRNA and other RNA at 36–48 h. However, there are still dozens of mRNAs and lincRNAs that are down- and upregulated during the different stages of endoderm differentiation. Specific miRNAs are known to play roles in maintaining pluripotency but also promote stem cell differentiation. hESCs express various specific miRNA signatures^48–51^ that are likely to support the pluripotent state and block differentiation. On the other hand, it has also been found that definitive endoderm cells can be distinguished from pluripotent hESCs by its unique miRNA profile, which consists of dozens of downregulated and upregulated miRNAs, and several miRNAs contribute to endoderm specification^52–57^.

Interestingly, tissue-specific super-enhancers start to be established before cell division to be fully established after division. Thus, transcription factors for which RNA expression is induced before division become fully active in the following cell cycle. Alternatively, some of these proteins could also act as mitotic bookmarking transcription factors to programme the identity of the newly created daughter cells before cellular division^58^. Similar mechanisms seem to exist between the first division-producing mesendoderm cells and the second division-producing endoderm cells. However, the second division is mainly associated with a reduction in the number of super-enhancers. Thus, differentiation could start with the establishment of a diverse set of tissue-specific super-enhancers which are then successively selected or discarded to enable the production of more committed cells (either endoderm or mesoderm). Further functional studies will help to identify the mechanism by which mesendoderm and endoderm transcriptional networks could achieve divergent activities.

The JNK–JUN pathway inhibits exit from the pluripotent state via JUN binding on pluripotency enhancers with OCT4, NANOG, SMAD2 and SMAD3^59^. By digital genomic footprinting, cis-regulatory elements related to the MAPK signalling pathway were identified to participate in the closure of the pluripotency network. Our results suggest that the AP-1 complex is likely to play a key part in the establishment and resolution of the mesendodermal network toward endoderm. We found AP-1 complex-associated TFs as the best predictors of chromatin accessibility change between day 1 and day 2, and proved experimentally that p38/MAPK is indispensable for endoderm specification by using small-molecule inhibitors. Thus, control of AP-1 activity by p38/MAPK, which is likely activated by the addition of FGF and PI3Kinase inhibition in our culture system, is essential for the first initial differentiation. Co-immunoprecipitation data and ChIP-qPCR indicated the binding of SMAD2/3 to NANOG, EOMES, GATA4 and AP-1 transcription factors FOSL2/JUN, and their participation in the regulation of mesendoderm, definitive endoderm as well as mesoderm. The induction of different transcription factors during successive cell cycles could be a mechanism that maintains cellular memory with the help of the expression of pioneer TFs. Our findings suggest a mechanism by which SMAD2/3, together with other transcription factors, forms a feed-forward circuitry that directs gradual stem cell differentiation, and could help to explain molecular mechanisms that are involved in the cell-autonomous formation of complex tissues. Transient AP-1-bound enhancers have been found during Oct4/Sox2/Klf4/cMyc (OSKM)-mediated cell reprogramming, linked to the extinction of the somatic transcriptional network^60^. Thus, AP-1 could have a broad function beyond early human development to eliminate chromatin roadblocks and facilitate cell-state transitions.

Importantly, other transcription factors expressed in hPSCs, such as NRF1, TFAP2C, TP53^61^ and CTCF, seem to have a key function in the early induction of mesendoderm specification. CTCF is not only pivotal for the orchestration of topologically associated domains and chromatin loops, but also it displays cell-cycle dependent DNA-binding^62–64^. NRF1 is a methylation-sensitive TF that could behave as a pioneer factor during early differentiation^65^, while TFAP2C could act as a settler TF^66^ binding all accessible sites before the first cell division. Moreover, TFAP2C is involved in chromatin accessibility dynamics in early ectoderm differentiation from hPSCs^67^. Thus, the mechanisms uncovered by our study could also apply to alternative germ layers. Nonetheless, further investigation will be necessary to understand the functions and the interplays of these different factors in mesendoderm specification and during each cell cycle leading to endoderm cells.

Our results also underline the importance to include the cell cycle effect in epigenetic and transcriptomic analyses. Our ChIP-seq profiling showed that during the cell-identity transition, H3K27ac presents its highest increase at 36 h, resulting in the emergence of novel super-enhancers after the first cell division. However, most of them are lost at day 3 and thus could not be detected in previous studies using unsynchronised cells^5^. Furthermore, adding a cell division dimension to our analyses allowed to order molecular events that successively lead to changes in cellular identity. Of note, some of the mechanisms uncovered are likely to be conserved in vivo. As an example, the formation of super-enhancers and fate restriction by chromatin closure are in agreement with recent studies in mouse post-implantation embryos^68^. These regulatory mechanisms shaping the epigenome are likely to be relevant not only for developmental processes but also for other stem cells involved in normal homoeostasis and diseases such as cancer^69^.

Our experiments blocking the cell division with nocodazole^23^ showed that, if the cell division is blocked, this reduces the induction of later developmental markers and moderately slows down the differentiation. These experiments provide a first insight into the coordination of cell divisions and endoderm differentiation. However, further experiments and research dedicated to this topic are needed to sufficiently characterise the possible functional interdependence and precise mechanisms between cell cycle, cell division and differentiation.

One of the long-term goals in biomedicine is to use hPSCs for differentiating them into functional cell types such as pancreatic beta-cells for biomedical applications, but the low efficiency of cell differentiation has remained a challenge. Cell synchronisation or cell cycle manipulation can add value in patient-specific hiPSCs that have lower than optimal differentiation capacity to a certain germ layer or cell type^70,71^. The impact of using synchronised differentiation of cells could be particularly relevant for organoid systems differentiating in 3D conditions. The synchronisation could impact cell–cell signalling and extracellular signalling pathways that could be influenced by the cell cycle stage due to its effect on gene expression dynamics, the abundance of transmembrane receptors or secreted ligands in the 3D microenvironment, as well as the size of the cells and their division, that can have an effect on a cell to cell contact inhibition. This could impact the spontaneous self-organisation of cells and tissue morphology.

To examine the practical utility of our findings, we differentiated hPSCs to endoderm and further to pancreatic lineage by inducing the activity of p38-MAPK signalling with sorbitol during the endoderm specification stage. p38-MAPK signalling induction improved definitive endoderm formation and increased the efficiency of pancreatic insulin-producing beta-cell differentiation. Hence, our findings have practical significance for the more efficient generation of hIPSC-derived cell types for biomedical applications. This could be particularly useful in regenerative medicine and cell replacement therapies by differentiating patient-derived iPSCs to functional cell types such as insulin-producing beta-cells for Type I diabetes patients.

## Methods

### Cell culture of hESCs and FUCCI-hESCs lines

hESCs (H9 from WiCell) were grown in defined culture conditions as described previously^72^. H9 cells were passaged weekly using collagenase IV and maintained in a chemically defined medium (CDM) supplemented with Activin A (10 ng/ml) and FGF2 (12 ng/ml). Pluripotent cells were maintained in Chemically Defined Media with BSA (CDM-BSA) supplemented with 10 ng/ml recombinant human Activin A and 12 ng/ml recombinant human FGF2 (both from Dr. Marko Hyvonen, Dept. of Biochemistry, University of Cambridge). Cells were passaged every 4–6 days with collagenase IV as clumps of 50–100 cells and dispensed at a density of 100−150 clumps/cm^2^. The culture media was replaced 48 h after the split and then every 24 h. Alternative culture conditions were used to maintain hPSCs used to study the role of ERK5-MAPK, p38-MAPK and JNK-MAPK. In sum, H9 and hIPSC lines FSPS13B and cA1ATD were routinely maintained on Vitronectin (StemCell Technologies)-coated plates in Essential 8 (E8) medium (Life technologies). Cells were passaged every 5–7 days using 0.5 µM EDTA and plated onto fresh vitronectin-coated plates in E8 medium. The medium was refreshed every day. This change corresponds to the modification of protocols in our lab and has no influence on experimental outcomes. The generation of FUCCI-hESC lines has been described in ref. ^18^ and are based on the FUCCI system described in ref. ^19^.

### In vitro differentiation of hESCs

FUCCI-hESCs were differentiated into endoderm as described previously^9^. Differentiation into endoderm was performed for up to 72 h with a combination of cytokines as described in refs. ^18,25^. For cells sorted by FACS, the cells were collected and immediately placed into the endoderm differentiation media. Endoderm specification was performed in CDM with polyvinyl Alcohol (CDM-PVA) prepared without insulin and supplemented with 50 ng/ml FGF2, 1 µM Ly-294002 (Promega), 100 ng/ml Activin A and 10 ng/ml BMP4 (R&D) for 3 days. Alternatively, and for cells grown in E8 medium, H9, FSPS13B or cA1ATD cells were plated as single cells onto gelatin/MEF-coated plates in E8 medium supplemented with 10 µM Y-27632. The medium was refreshed the next day. Chemically defined media with polyvinyl alcohol (CDM-PVA) containing 100 ng/ml recombinant Activin A (CSCR, University of Cambridge), 80 ng/ml FGF2 (R&D Systems), 10 ng/ml BMP4 (CSCR, University of Cambridge), 10 μM LY29004 (Promega) and 3 μM CHIR99021 (Selleck Chemicals) was applied to the cells for 24 h. The media was then replaced with fresh CDM-PVA supplemented with 100 ng/ml recombinant Activin A (CSCR, University of Cambridge), 80 ng/ml FGF2 (R&D Systems), 10 ng/ml BMP4 (CSCR, University of Cambridge) and 10 μM LY29004 (Promega). The next day, the media was removed and RPMI media supplemented with 1X B27 (Lifetech), 100 ng/ml Activin A, 80 ng/ml FGF2 and 1X non-essential amino acids (Lifetech) was added to the cells. To investigate the roles of ERK5-MAPK, p38-MAPK and JNK-MAPK, the endoderm differentiation media was supplemented with 10 µM U0126-EtOH, 10 µM SB203580 or 1 µM JNK-IN-8 respectively. To investigate the effect of AP-1 activation on cell differentiation, we placed cells in an embryoid body medium based on E8 in ultra-low binding plates (Corning). For the activation of the AP-1 pathway, (50 ng/ml) PDGF-BB (Peprotech) and 100 nM 12-*O*-tetradecanoylphorbol-13-acetate (Merck) were used in the embryoid body medium and incubated for 3 days.

### Sorbitol treatment during differentiation

Cells were treated with 200 mM Sorbitol (Merck) during 24 to 72 h of endoderm differentiation to activate the p38-MAPK signalling pathway.

### Cell sorting by FACS

FACS on FUCCI-hESCs was performed as described before^18,19^. hESCs were washed with PBS and detached from the plate by incubating them for 10 min at 37 °C in cell dissociation buffer (Gibco). Cells were then washed with a cold filter and sterilised 1% BSA in PBS, before incubating cells in PBS 1% BSA with Tra-1-60 primary antibody (1:100) and Alexa Fluor 647 donkey α-mouse secondary antibody (1:1000) on ice for 20 min in the dark with occasional gentle mixing. The cells were then washed once with at least 50x pellet volume PBS 1% BSA, resuspended gently in 3 ml sterile hESC maintenance media, and subjected to cell sorting by gating Tra-1-60+ cells according to the mAG/mKO2 FUCCI signals. The cell sorting was performed with a Beckman Coulter MoFlo MLS high-speed cell sorter by using parameters described previously^18^, and the cells were sorted directly into collection tubes with 2 ml hESC maintenance media. After sorting, the cells were pelleted and placed in endoderm differentiation media. The media was changed every 24 h and samples were collected at different time points for subsequent processing for RNA-seq, histone mark ChIP-seq and ATAC-seq. A representative cell sorting and gating strategy in FUCCI-expressing cells is shown in Supplementary Fig. 7.

### Immunostaining

The immunostaining method has been described previously^18,25,73^. Cells were fixed for 20 min at 4 °C in PBS 4% PFA (electron microscopy grade), rinsed three times with PBS, and blocked and permeabilised at the same time for 30 min at room temperature using PBS with 10% Donkey Serum (Biorad) and 0.1% Triton X-100 (Sigma). Incubation with primary antibodies (Supplementary Information—Supplementary Table 1) diluted in PBS 1% Donkey Serum 0.1% Triton X-100 was performed overnight at 4 °C. Samples were washed three times with PBS, and then incubated with Alexa Fluor secondary antibodies (Supplementary Information—Supplementary Table 1) for 1 h at room temperature protected from light. Cells were finally washed three times with PBS, and Hoechst (Sigma) was added to the first wash to stain nuclei. Images were acquired using an LSM 700 confocal microscope (Leica).

### Flow cytometry

Single-cell suspensions were prepared by incubation in Cell Dissociation Buffer (Gibco) for 10 min at 37° followed by gentle pipetting. Cells were fixed in 4% PFA for 20 min at 4 °C. This was followed by permeabilization and blocking with 10% serum + 0.1% Triton X-100 in PBS for 30 min at RT and incubation with the primary antibody in 1% serum + 0.1% Triton X-100 for 2 h at 4 °C. After washing the samples three times with PBS, they were incubated with a secondary antibody for 2 h at 4 °C, washed three times with PBS and analysed by flow cytometry. Flow cytometry was performed using a Cyan ADP flow cytometer and at least 20,000 events were recorded. Data were analysed by FlowJo software. Cell cycle distribution was analysed by Click-It EdU incorporation Kit (Invitrogen) according to the manufacturer’s guidelines.

### RNA-seq experiments

Samples for RNA sequencing were collected at different time points (0, 12, 24, 36, 48, 60 and 72 h) from FUCCI-hESCs differentiated to endoderm. The libraries for RNA-seq were generated by the Wellcome Sanger Institute Illumina Bespoke Sequencing Facility and sequencing was performed onsite. The libraries were generated at a library fragment size between 100 bp to 1 kb with Stranded RNA-seq Standard with Oligo dT pulldown. The samples were multiplexed and analysed by Illumina Hiseq V4 with a paired-end read length PE75. All samples were amplified with a standard ten PCR cycle before sequencing. Samples were distributed equally across sequencing lanes and a total of 2,037,382,345 mapped reads with MAPQ ≥10 were obtained after sequencing using Illumina HiSeq 2000 (97,018,207 reads/sample on average).

### ChIP-seq experiments

ChIP-seq was performed using FUCCI-Human Embryonic Stem Cells (FUCCI-hESCs, H9 from WiCell) in a modified endoderm differentiation protocol (see details below). Cells were grown in defined culture conditions as described previously^72^. Pluripotent cells were maintained in Chemically Defined Media with BSA (CDM-BSA) supplemented with 10 ng/mL recombinant Activin A and 12 ng/mL recombinant FGF2 (both from Dr. Marko Hyvonen, Dept. of Biochemistry, University of Cambridge) on 0.1% Gelatin and MEF media coated plates. Cells were passaged every 4–6 days with collagenase IV as clumps of 50–100 cells. The culture media was replaced 48 h after the split and then every 24 h.

The generation of FUCCI-hESC lines is based on the FUCCI system^18,19^. hESCs were differentiated into endoderm as previously described^9^. Following FACS sorting, Early G1 (EG1) cells were collected and immediately placed into the endoderm differentiation media and time points were collected at 0, 12, 24, 36, 48 and 72 h. Endoderm specification was performed in CDM with polyvynilic acid (CDM-PVA) supplemented with 20 ng/mL FGF2, 10 µM Ly-294002 (Promega), 100 ng/mL Activin A and 10 ng/mL BMP4 (R&D). We performed ChIP-seq for various histone marks (H3K4me3, H3K27me3, H3K4me1, H3K27ac, H3K36me3) (see Supplementary Information—Supplementary Table 1 for antibodies), on two biological replicates per condition^25^, except at 36 h time point, where only one replicate was obtained, and we could not generate H3K27me3 (H3K27me3 for 36 h sample failed, but we do expect a reduced number of changes based in comparison between 24 and 48 h). At the end of the ChIP protocol, fragments between 100 and 400 bp were used to prepare barcoded sequencing libraries. Ten nanograms of input material for each condition were also used for library preparation and later used as a control during peak calling. The libraries were generated by performing eight PCR cycles for all samples. Equimolar amounts of each library were pooled, and this multiplexed library was diluted to 8 pM before sequencing using an Illumina HiSeq 2000 with 75 bp paired-end reads.

### ChIP-qPCR experiments

The ChIP experiment for ChIP-qPCR was performed as described previously^25^. At the end of the ChIP protocol, samples were used for qPCR analysis with primers described in Supplementary Information—Supplementary Table 3.

### ATAC-seq experiments

hESCs were sorted to the early G1 phase and plated at 200,000 cells per well in 12-well plates with 0.5 ml endoderm differentiation media. After differentiating the cells for a range of time points, the cells were washed once with PBS, collected in Cell Dissociation Buffer (Gibco 13150-016) and centrifuged at 300 g for 3 min. The cell pellets were then resuspended in 2 ml of 4 °C PBS and counted by haemocytometer for using 100,000 cells in the subsequent step. Cells were centrifuged at 300 × *g* for 3 min, the supernatant aspirated, the cell pellet resuspended in 150 ul of isotonic lysis buffer (10 mM Tris-HCl pH 7.5, 3 mM CaCl, 2 mM MgCl2, 0.32 M sucrose and protease inhibitors, Roche), and incubated for 12 min on ice. Triton X-100 from a 10% stock was then added at a final concentration of 0.5%, the samples were vortexed briefly and incubated on ice for 6 min. The samples were centrifuged for 5 min at 400 × *g* at 4 °C, and the cytoplasmic fraction was removed from the nuclear pellet. The samples were resuspended gently in 1 ml of isotonic lysis buffer and transferred to a fresh 1.5 ml Eppendorf tube. The nuclei were centrifuged at 1500 × *g* for 3 min at 4 °C and the supernatant was aspirated thoroughly from the nuclear pellet. This step was immediately followed by tagmentation (Nextera DNA Sample Preparation Kit for 24 Samples, FC-121-1030) by resuspending each sample in 50 µl Nextera mastermix (25 µl TD buffer, 20 µl of water and 5 µl of TDE1 per reaction). The nuclear pellet was resuspended thoroughly by pipetting and incubated at 37 °C for 30 min. The reaction was stopped with 250 µL of buffer PB from the Qiagen PCR purification kit, followed by Qiagen PCR clean-up protocol using MinElute columns and eluting each sample in 11.5 µl buffer EB. For the control sample, the nuclear pellet was subjected to genomic DNA isolation with GenElute Mammalian Genomic DNA Miniprep Kit (Sigma, G1N70) according to the manufacturer’s protocol, and the purified genomic DNA was thereafter immediately used for tagmentation as for other ATAC-seq samples. Next, a PCR reaction (for all samples including the control sample) was performed with the following constituents: 10 µl template from tagmentation, 2.5 µl I7 primer (Nextera® Index Kit with 24 Indices for 96 Samples, FC-121-1011), 2.5 µl I5 primer, 2.5 µl Nextera cocktail and 7.5 µl Nextera PCR mastermix. The PCR settings were as follows: initial denaturation at 98 °C for 30 s, then 12 cycles of 98 °C for 10 s, primer annealing at 63 °C for 30 s and elongation at 72 °C for 3 min, which was followed by a final elongation at 72 °C for 3 min and holding at 10 °C. After completing the PCR, the sample volumes were increased to 50 µl by adding Qiagen EB buffer from the PCR purification kit. The PCR primers were removed with 1 ×0.9:1 SPRI beads (Beckman Coulter, Cat no. A63880) according to the manufacturer’s protocol and samples were eluted in 20 µl. DNA size selection was performed as follows: the samples were run on a 1% agarose gel in TAE Buffer at 90 V for 25 min. The DNA was cut within the range of 150 bp to 1 kb and purified by Qiagen Gel Extraction Kit with MinElute columns, by eluting in 20 µL Qiagen buffer EB. 1 µl of the samples were run on Agilent HS Bioanalyzer HS for confirming the size selection of the ATAC libraries. ATAC-sequencing was performed by Illumina HiSeq 2000 sequencing with 75 bp PE for obtaining 460,130,600 million reads per library on average (5,981,697,796 in total for 0, 12, 24, 36, 48 and 72 h samples in duplicates plus a control sample).

### Quantitative real-time PCR

Media was removed and cells were washed once with DPBS (Life Technologies) before 300 µl of RNA lysis buffer was added. RNA was extracted using the GenElute Mammalian Total RNA Miniprep Kit (Sigma-Aldrich) per the manufacturer’s instructions. About 500 ng RNA was reverse-transcribed using random primers (Promega), dNTPs (Promega), RNAseOUT (Invitrogen), DTT (Invitrogen) and SuperScript II (Invitrogen). QPCR reactions were made up using 2x KAPA SYBR Fast qPCR Master Mix kit (Kapa Biosystems), 4.2 ul of 30x diluted cDNA and 200 nM of forward and reverse primers. Samples were run in the QuantStudio 12 K Flex real-time PCR system machine and a 384-well plate and analysed using the delta-delta cycle threshold (Ct) method normalised to the housekeeping gene, ACTB. Primer sequences can be found in Supplementary Information—Supplementary Table 2 and in ref. ^18^.

### Immunofluorescence analysis of synchronised cells following nocodazole treatment

Related to Supplementary Fig. 4c, blocking of hESCs in the G2/M phase of the cell cycle is reversible, and cells can progress into the following G1 phase after withdrawing Nocodazole from the culture medium^23^. Accordingly, hESCs were synchronised in the G2/M phases of the cell cycle as described in ref. ^74^, including some minor modifications. To obtain better survival of cells during the synchronisation and differentiation steps, small colonies were plated onto coverslips coated with Vitronectin XF (Stem Cells Technologies, 07180) and allowed to grow for 2–3 days. Robust medium-size colonies were treated with Nocodazole (Sigma-Aldrich, Catalogue number M1404-2MG, 100 ng/ml) for 16 h before the induction of differentiation. Following synchronisation, cells were gently washed twice with fresh medium and immediately transferred into endoderm differentiation medium. At the required times, cells were fixed with paraformaldehyde 3.7% for 10 min. at room temperature and submitted to immunofluorescence staining. Subsequently, cells were permeabilised in 0.25% Triton X-100 in PBS and blocked 0.5% bovine serum albumin (BSA) in PBS. Cells were stained with the following antibodies for 1 h at room temperature: anti-OCT4 (Santa Cruz Biotechnology, sc5279 [1:200]), anti-T (R&D Systems, AF2085 [1:200]), anti-TFAP2C (Santa Cruz Biotechnology, sc12762 [1:100]), anti-NRF1 (Abcam, ab55744 [1:50]) and anti-SOX17 (R&D Systems, AF1924 [1:200]). Nuclear DNA was counterstained with DAPI (0.1 μg/ml). Secondary antibodies conjugated with Alexa Fluor 488, Alexa Fluor 568 or Alexa 647 (1:1000; Molecular Probes/Invitrogen) were incubated for 1 h in a humidity chamber, and at room temperature. Cells were washed three times with PBS-BSA after every antibody incubation step. Finally, coverslips were mounted onto glass slides using ProLong Gold antifade mounting medium (Thermo Fisher Scientific, P36930). Cells were analysed under a confocal microscope the next day.

### Protein co-immunoprecipitation

Cells were harvested with trypsin and washed twice with cold PBS. For cytoplasmic lysis, cells were suspended in five times packed cell volume (1 µl PCV = 10^6^ cells) equivalent of Isotonic Lysis Buffer (10 mM Tris-HCl, pH 7.5, 3 mM CaCl, 2 mM MgCl_2_, 0.32 M sucrose, Complete protease inhibitors and phosphatase inhibitors), and incubated for 12 min on ice. Triton X-100 was added to a final concentration of 0.3% and incubated for 3 min. The suspension was centrifuged for 5 min at 1500 rpm at 4 °C and the supernatant (cytoplasmic fraction) was transferred to a fresh chilled tube. For nuclear lysis, nuclear pellets were resuspended in 2x PCV Nuclear Lysis Buffer + Triton X-100 (50 mM Tris-HCl, pH 7.5, 100 mM NaCl, 50 mM KCl, 2 mM MgCl_2,_ 1 mM EDTA, 10% Glycerol, 0.3% Triton X-100, Complete protease inhibitors and phosphatase inhibitors) and dounce homogenised. The samples were incubated with gentle agitation for 30 min at 4 °C and then centrifuged with a Ti 70.1 rotor at 22,000 rpm for 30 min at 4 °C or with a Ti 45 rotor for 30 min at 20,000 rpm at 4 °C. The chromatin pellets were dounce homogenised in 2x PCV Nuclear Lysis Buffer + Triton X-100 and Benzonase until the pellets gave much less resistance. The samples were incubated at RT for 30 min and centrifuged with either a Ti 70.1 rotor for 30 min at 22,000 rpm at 4 °C or with a Ti 45 rotor for 30 min at 20,000 rpm at 4 °C. Samples were incubated with 5 µg of cross-linked antibodies for 12 h at 4 °C. Beads were washed five times with ten bead volumes of Nuclear Lysis Buffer and eluted in SDS western blotting buffer (30 mM Tris pH 6.8, 10% Glycerol, 2% SDS, 0.36 M beta-mercaptoethanol (Sigma), 0.02% bromophenol blue) by heating at 90 °C for 5 min. Samples were analysed by standard western blotting techniques.

### Pancreatic differentiation

Pancreatic differentiation was carried out as follows, as described in ref. ^18^. Daily media changes were made during the entire differentiation protocol. After endoderm differentiation, cells were cultured in Advanced DMEM (Invitrogen) supplemented with SB431542 (10 μM; Tocris), FGF10 (50 ng/ml; AutogenBioclear), all-trans retinoic acid (RA, 2 μM; Sigma) and Noggin (50 ng/ml; R&D Systems) for 3 days. Cells were then cultured in Advanced DMEM + human FGF10 (50 ng/ml; AutogenBioclear), all-trans retinoic acid (RA, 2 μM; Sigma), KAAD-cyclopamine (0.25 μM; Toronto Research Chemicals) and Noggin (50 ng/ml; R&D Systems) for 3 days. Next, cells were cultured in human KGF (50 ng/ml; R&D Systems) for 3 days. For maturation of pancreatic progenitors, cells were grown in Advanced DMEM + 1% vol/vol B27 and DAPT (1 mM) for 3 days and for 3 additional days in Advanced DMEM + 1% vol/vol B27.

### LZTS1 overexpression

For overexpression of LZTS1, we used SP-dCas9-VPR with a doxycycline-inducible expression system as described previously^75,76^. PB-TRE-dCas9-VPR was a gift from George Church (Addgene plasmid # 63800; http://n2t.net/addgene:63800; RRID:Addgene_63800). gRNA_Cloning Vector was a gift from George Church (Addgene plasmid # 41824; http://n2t.net/addgene:41824; RRID:Addgene_41824). gRNA sequences were obtained from the GenScript gRNA database.

### LZTS1 effects on endoderm and pancreatic differentiation

LZTS1 overexpression effects on endoderm were analysed by qPCR and western blot on samples collected at different time points during endoderm differentiation. LZTS1 overexpression effect on pancreatic beta-cells was analysed on cells that were differentiated as described previously^18^. qPCR and western blotting were performed on samples as described under the specific method sections. 2 mM Doxycyclin was used for inducible induction of LZTS1. Western blot band intensities were measured using ImageJ (https://imagej.nih.gov/ij/index.html).

### Nocodazole treatment for qPCR and histone ChIP-qPCR

Cells were treated with Nocodazole during endoderm differentiation up to the 48 h time point, and samples were collected for qPCR analysis and histone H3K4me3 and H3K27me3 ChIP-qPCR of endoderm marker expression at different time points starting from 0, 24, 36 and 72 h from the start of endoderm differentiation. qPCR and ChIP-qPCR were performed as described in the Quantitative real-time PCR and ChIP-qPCR experiments section.

### EOMES inducible knockdown (iKD) during endoderm differentiation

Inducible knockdown of EOMES was performed by using a dox-inducible CRISPR interference (CRISPRi) knock-in construct that was first targeted to the AAVS1 locus to create a stable hESC line as described in ref. ^77^. gRNA sequences for EOMES knockdown were obtained from the GenScript gRNA database and cloned into the gRNA construct according to the protocol from ref. ^72^. 2 mM Doxycyclin (Dox) was used for dCas9-KRAB mediated inducible knockdown of EOMES during endoderm differentiation. Dox was added 12 h after the initiation of endoderm differentiation and added together with fresh endoderm differentiation media every 3 days. Cells were cultured beyond the normal definitive endoderm time point from day 3 until day 12 in order to estimate the effects of EOMES iKD endoderm and neuroectoderm marker expression. Samples were collected every 3 days for gene expression analyses by qPCR. Normal neuroectoderm differentiation was performed in parallel to compare marker expression. pAAVS1-NDi-CRISPRi (Gen2) was a gift from Bruce Conklin (Addgene plasmid # 73498; http://n2t.net/addgene:73498; RRID:Addgene_73498). pgRNA-CKB was a gift from Bruce Conklin (Addgene plasmid # 73501; http://n2t.net/addgene:73501; RRID:Addgene_73501).

### RNA-seq data analysis

Reads were mapped to the human genome (GRCh38.15) using TopHat v2.0.13^78^ with the following options: '--library-type fr-firststrand', '--mate-inner-dist 100 --no-coverage-search --microexon-search' and '—transcriptome-index' with a TopHat transcript index built from ensembl_76_transcriptome-GRCh38_15.gtf. Reads with Mapping Quality Values <10 were filtered out with samtools^79^. featureCounts was used on paired-end reads to count fragments in annotated gene features, with parameters ‘-p -C -T 8 -t exon -g gene_id’^80^. DESeq2 was used for differential gene expression analysis between samples, requiring at least a 1.5-fold expression change and a Benjamini–Hochberg adjusted *P* value smaller than 0.01^81^ for a gene to be deemed as differentially expressed. The function ‘rpkm’ in the R/Bioconductor package edgeR^82^ was used with default parameters to normalise count gene expression. Raw bedGraphs were normalised per million mapped reads in the library per library size in all samples^83^. Spearman’s correlation *ρ* values were calculated for FPKM expression values of genes expressed at more than 5.0 FPKM in at least one of the samples under comparison. Hierarchical clustering of *ρ* values clustered all the triplicates in each condition together (Supplementary Fig. 1h). PCA implemented in DESeq2 was performed using all datasets (Supplementary Fig. 1i). Other bioinformatics analyses were carried out following standard procedures^83^.

### *K*-means clustering in RNA-seq time course

Model-based optimal number of clusters *K* that minimised Bayesian Information Criterion (BIC) was considered for 6317 differentially expressed protein-coding genes. The number of clusters *K* = 13 was selected as the one that minimised the BIC using the function ‘mclust’ in the R package ‘mclust’. To smooth the data for representing the curves in Fig. 1h, we used the functional data analysis R package 'fda' v2.4.4. First, we represented data values using 5 B-spline basis functions located at 0, 12, 24, 36, 48, 60, 72 h without roughness penalties in the second derivative (*λ* = 0). We used the functions ‘create.bspline.basis’ and ‘smooth.fd’ over the interval 0–72 h. Then, we evaluated the mean and the s.d. of the functional data objects using the R functions ‘mean.fd’ and ‘sd.fd’.

### Cell-cycle phase scoring in single-cell RNA-seq

Processed single-cell count data in ref. ^21^ was downloaded from Zenodo: https://zenodo.org/record/3625024#.Xil-0y2cZ0s. Cell cycle scoring and classification was performed with the R package Seurat v3.0.2^84^.

### ChIP-seq data analysis

Reads were mapped to the human GRCh38 reference assembly using BWA^85^. Only reads with mapping quality score ≥10 and aligned to autosomal and sex chromosomes were kept for further processing. Peak calling analysis^86^ was performed using PeakRanger^87^, and only the peaks that were reproducible at an *FDR* ≤ 0.05 in two biological replicates were selected for further processing. Peak calling was done using appropriate controls with the tool peakranger v1.18 in modes *ranger* (H3K4me3, H3K27ac; ‘-l 316 -b 200 -q 0.05’), *ccat* (H3K27me3; ‘-l 316 --win_size 1000 --win_step 100 --min_count 70 --min_score 7 -q 0.05’) and *bcp* (H3K4me1, H3K36me3; ‘-l 316’). Adjacent peak regions closer than 40 bp were merged using the BEDTools suite^88^, and those overlapping ENCODE blacklisted regions were filtered out (ENCODE Excludable Mappability Regions^89^). bedGraph format files were produced for each sample using BEDTools 2.17.0^88^. The reads mapped at both DNA strands from 5′ to 3′ direction were extended to a length of 316 bp, and the read count at each genomic position was normalised to the library size and per million reads (multiplying every value by ‘1,000,000/number_of_mapped_reads’). bedGraph files were converted to bigWig using UCSC tool bedGraphToBigWig, and are available for visualisation on the Biodalliance genome viewer^90^ at: http://ngs.sanger.ac.uk/production/endoderm.

### Differential peak calling in histone modification ChIP-seq

G-tests implemented in diffReps^91^ were used to detect differential histone modification regions. Because we have only one replicate at 36 h, we decided to use G-tests instead of Negative Binomial tests for all the comparisons, as recommended in ref. ^91^. All input samples were merged and used as background control. Differential histone modifications regions not overlapping at least 1 bp with significant chromatin marks previously detected during peak calling at least in one of the conditions under comparison were removed. Regions were ranked by their adjusted *P* value and reported as differentially enriched only if the absolute FC ≥2, and Benjamini–Hochberg corrected *P* value ≤ 0.01. Genes in a 10 kb window of the regions were reported.

### ATAC-seq data analysis

A total of 5,981,697,796 PE reads (75 bp) were sequenced using Illumina HiSeq 2000, which includes one deeply sequenced control sample of 369,590,751 reads. BWA v0.7.12^85^ with parameters ‘mem -t 16 -p -T 0’ was used for read alignment against hg38 (GRCh38.15) reference assembly of the human genome. Aligned reads were retained if MAPQ ≥5. Only autosomal and sex chromosomes were retained. Mitochondrial contamination (% reads) was found proportional to the differentiation stage; high (~80%) in hESCs and low in definitive endoderm (~25%). Peak calling against the control sample was performed using JAMM v1.0.7.1^92^, with parameters ‘-m normal -r region -f 1,1,1 –b 140’. Duplicates were removed at this stage as in^29^ to improve peak calling, same as recommended in ChIP-seq data analysis^86^. To select the number *n* of reproducible peaks at *IDR* ≤ 0.05, Irreproducible Discovery Rate (IDR) analysis^93^ was performed on JAMM’s ‘filtered’ peaks of individual replicates using JAMM’s peak scores S_p_ (1), which are defined as:

1 $${S}_{p}={\mu }_{{{{{{{\mathrm{ns}}}}}}}}\times \left(-{\log }_{10}P\right)$$

where *μ*_ns_ is the mean peak background normalised signal, and *P* is the Benjamini–Hochberg corrected *P* value of Mann–Whitney *U* non-parametric tests. A number of peaks equal to the minimum number of peaks in one of the replicates were submitted for IDR analysis. Top *n* peaks in JAMM’s replicate integration (pooled replicates) method were then selected as a highly confident set. Normalised signal tracks were built after extending each read to estimate an average fragment size of 140 bp. We generated a consensus-merged list of 253,618 peak regions, filtered out those overlapping the human ENCODE blacklisted genomic regions (https://sites.google.com/site/anshulkundaje/projects/blacklists), and considered a final set of 253,349 open chromatin regions for further analyses. Processed ATAC-seq data is freely available at http://ngs.sanger.ac.uk/production/endoderm.

### Differential open chromatin region analysis in ATAC-seq

To statistically identify regions of differential open chromatin in ATAC assays, diffNGS^94^ was used (http://github.com/pmb59/diffNGS). We performed Hotelling’s *T*^2^ tests on the functional principal component scores to identify significant differences across conditions. Genomic regions were declared significant for Bonferroni adjusted *P* value ≤10^−4^ and absolute fold change (FC) ≥2.0 in a set of aggregated regions of those under comparison. 31 bins were used for signal extraction in the R package ‘genomation’^95^ and 10 equidistant *B*-splines bases were used for functional principal component analysis. Regions were then ranked then by the scores (2)):

2 $${S}_{d}=-\log \left({{{{{{\mathrm{adj}}}}}}}.P\right)\times {{{{{{\mathrm{FC}}}}}}}\times \sqrt{{{{{{{\mathrm{Baseline}}}}}}}}$$

(Data available here: http://ngs.sanger.ac.uk/production/endoderm/), where baseline is defined as ½*(Avg. normalised signal in time point 1+Avg. normalised signal in time point 2). Genes were associated with peaks if the former were in a region 10 kb upstream or downstream from differential open chromatin regions. Open chromatin regions with no change at any stage were classified as ‘invariant’.

### Motif analysis of DNA sequences in differential ATAC-seq regions

Motif enrichment analysis was performed with HOMER (v4.8.3), using the function ‘scrambleFasta.pl’ to create a set of background frequencies (with the same number of sequences, as recommended). We scored known motifs for enrichment in the FASTA files using the function ‘known’, and motifs from the CIS-BP database^96^ (3059 PWMs; http://cisbp.ccbr.utoronto.ca). Occurrences were ranked and a scatter plot was generated using their Log(*P* value) and Log_2_(enrichment ratio) for TF motifs associated to genes expressed at least 1.0 FPKM (mean across triplicates) in the earliest time point of the two consecutives being analysed. TF_Name was associated to PWMs using the CISBP information file TF_Information.txt (of directly determined motifs and best-inferred motifs).

### Pearson correlation between RNA-seq and ATAC-seq

Pearson’s product-moment correlation was calculated in R using the function cor.test (alternative hypothesis: true correlation is not equal to 0). Log_2_ fold changes obtained in DESeq2 for protein-coding genes were used. To obtain the Fold change in ATAC-seq, maxima of normalised ATAC-seq signal in a region 10 kb upstream of the promoter of protein-coding genes was obtained with ScoreMatrixBin (genomation package^95^) in each ATAC-seq replicate of consecutive time points, and log_2_ of the fold change was computed.

### GO enrichment analysis in variant open chromatin regions

Ensembl gene IDs of protein_coding genes were converted to Entrez gene IDs using biomaRt (Ensembl 76). Gene Ontology (GO) enrichment analysis was performed using GOstats^97^, Hypergeometric Tests for GO term association (function ‘hyperGTest’) were run with BP ontology and *P* value cutoff = 0.001. This function computes hypergeometric *P* values for over- (or under-) representation of each GO term in the specified ontology among the GO annotations for the genes of interest.

### Identification of footprints of DNA-binding transcription factors

We used FootPrintMixture^98^ to detect footprints of TFs in ATAC-seq, adapting this tool to explicitly control for assay-specific sequence bias, which is fundamental for DNase-seq and ATAC-seq data analysis^99,100^. PWMs of ‘Directly determined or best-inferred motif’ for *Homo sapiens* were downloaded from CIS-BP database^96^, 3059 valid PWMs were used.

To adjust the sequence bias with a deproteinized ATAC control, we retrieved Tn5 transposition sites from the deproteinized ATAC control sample (328,759,008 mapped reads) after translating 5′-ends of the reads +4 bp for insertion on the forward strand, and −5 bp in the reverse strand^29^. Then we obtained 6-mers frequencies ±3 bp around the transposition site for *chr1* using Jellyfish^101^ count with parameters “-m 6 -s 4000 M -t 4”. Background 6-mer counts in the human genome were estimated using the ‘fasta-get-markov’ programme in the MEME Suite with a Markov Model of order *m* = 5. Then, the SeqBias values in FootprintMixture represent *k*-mer frequencies in the deproteinized sample divided by background frequencies.

Matches of the PWMs were obtained using FIMO^102^ with a cut-off of *P* < 10^−4^, then ranked, and motifs with at least 10k matches (up to a maximum of 500k matches) were submitted for the two-component mixture model in FootPrintMixture to infer TF binding ±25 bp around each motif. Extended motif occurrences that lie out of genomic regions in hg38 were not submitted to analysis. To get high-quality reproducible footprints, motif matches with footprint likelihood ratio (FLR) ≥10.0 in each biological replicate were classified as 'Bound'. TF footprints that were present in both replicates, and with at least 50 FPs for the PWM, were considered as predicted TF BSs. FLR mean of the replicates was calculated. For this calculation, only unique genomic regions were considered (for those palindromic sequences with a footprint reported in each strand, we obtained the mean FLR). Normalised average of Tn5 insertion densities for ‘Bound’ sites were plotted ±100 bp around motif using R and NucleoATAC^103^ function ‘pyatac ins’ for the whole genome, after normalising for the number of reads in the library. For visualisation purposes, reads in both replicates were merged. Next, we computed the protection scores (PS) for footprint predictions of a PWM as proposed in ref. ^104^.

Flanking regions were considered ±25 bp upstream/downstream of the motif for this calculation. Footprints with short DNA residency time are poorly detected and likely to be false positives. TFs with intermediate and long residency times have positive PS^104^. Footprints with a negative protection score and associated with genes with ≤1.0 FPKM (no expression) were removed.

We run also Wellington/pyDNase 0.2.4 for digital genomic footprinting, a non-motif centric footprint algorithm detector^105^ to search TF footprints between 4–30 bp (‘-A -fdr 0.1 --FDR_limit −4 --pv_cutoffs −4 -fp 4,30,1 -fdriter 500 --one_dimension’). As search space, we used the set of consensus open chromatin regions extended 50 bp upstream and downstream. BAM files of alignments for replicates were merged to achieve better coverage. A relaxed set of footprints was obtained at each time point (*P* ≤ 1e-4).

The final set of curated nonredundant footprints were considered as those with:

- FLR ≥10.0 (FootprintMixture), reproducible in both replicates.
- Protection score PS ≥0.
- CiSBP PWM is associated with only 1 TF.
- Expression of gene/TF ≥1.0 FPKM (from RNA-seq at the same stage).
- Only footprints with the highest FLR were kept for Overlapping footprints (distinct PWMs) for the same TF (in case of equal FLR, we took the first) to get a nonredundant set.
- Finally, only nonredundant footprints detected also by Wellington with a relaxed cut-off were kept.

### Differential genomic footprinting

Wellington_bootstrap/pyDNase v0.2.4^38^ was used to obtain differential footprints between consecutive time stages (http://pythonhosted.org/pyDNase). As a search space, a BED file with genomic locations was provided and consisted of the consensus open chromatin sites extended 50 bp on each side. BAM files of alignment for replicates were merged and evaluated by Wellington_bootstrap with parameters '-A -fp 4,30,1 -fdr 0.05 -fdrlimit −10 -fdriter 100'. Importantly, Wellington_bootstrap allows to control for different sequencing depths in the two samples^38^. A score cut-off of *S* ≥ 5 was used for differential footprints to be considered as such. The over-enrichment of the number of predictions for a TF was statistically assessed considering the proportion over the total footprints against its relative proportion in the differential list by applying Chi-squared tests. Benjamini–Hochberg (BH) multiple hypothesis testing correction was applied. In addition, bivariate genomic footprinting was performed with BaGFoot v.0.9.7^106^ over motifs of human transcription factors from the CiSBP database (http://cisbp.ccbr.utoronto.ca) to obtain global TF footprint pattern change between conditions.

### Peak annotation in ATAC-seq peaks and TF footprints

NIH PAVIS v.04-08-2016 was used for peak-to-gene annotation (GRCh38r76/hg38) of differential ATAC-seq peaks (https://manticore.niehs.nih.gov/pavis2/). Upstream Length: 10 kb, Downstream Length: 5 kb (rest of parameters under default configuration). TF footprints' unique regions were obtained using the function ‘merge’ in BEDTools before submitting to PAVIS.

### Overlap of differential chromatin accessibility regions between time points

Intersection and visualisation analysis of differential open chromatin regions was performed with Intervene version 0.6.5^107^.

### Chromatin accessibility comparison to non-mesendodermal data

We used previously published ATAC-sequencing data^28^ to represent non-mesendodermal/neuroectodermal samples. ATAC-seq datasets^28^ for hESC-derived ectoderm-directed differentiation cells after mesendoderm competence loss were analysed using the same pre-processing methods as in our datasets. We used the peaks called from 0 h (undifferentiated hPSCs) as reference regions and counted the reads mapped to these regions from the filtered BAM files using featureCounts v2.0.0^80^. Then, differential accessibility analysis was done using DESeq2 v1.36.0^81^. Significant differential accessible regions were defined as FDR-adjusted *P* value <0.05. Peaks were annotated to their closest TSS.

### Functional linear regression with a scalar response

Functional data analysis (FDA) has been applied in genomics to allow data analysis on continuous data^108^. Using a functional linear model^109^ instead of a multivariate linear model allows including in the model the spatial dependency between TF binding and chromatin change. The model used to predict chromatin accessibility change exerted by footprints of a transcription factor was (3):

3 $${y}_{i}={\alpha }_{0}+\mathop{\sum }\limits_{j=1}^{q}{x}_{{ij}}(t){\beta }_{j}(t){dt}+{\varepsilon }_{i}$$

where *y*_*i*_ is the chromatin change of region *i* between time point *t* and time point *t* + 1, *x*_*i*_*(t)* is a functional covariate (*q* = 1) defined by the footprinting signal, ${\alpha }_{0}$ is the intercept term, ${\beta }_{j}$ are the regression coefficient functions, and ${\varepsilon }_{i}$ is the independent and identically distributed (i.i.d.) error term. Only differential accessibility regions with a fold change FC >1.5 were considered. FC ≥|10| were removed and not considered to avoid outliers and 1000 bp were used around the centre of each ATAC-seq peak. Log_2_ (abs(FC)) was considered as the scalar variable *y*_*i*_ to be regressed. A maximum of 50,000 genomic regions, ranked by their abs(FC), were considered in the model. Putative TF binding sites were represented in the functional covariate as 1.0 (absence as 0.0) using five order-4 *B*-splines. The linear model was only considered when at least 15 footprints overlapped regions of interest, and only TFs with footprints in >50 of the selected regions were implemented in the model. Squared multiple correlation (*R*^2^) and *F*-ratio were used to assess the improvement of fit. *P* values were computed for the *F*-statistic using the ‘pf’ function in R. The R package ‘fda’ was used in these analyses (https://cran.r-project.org/web/packages/fda).

### Jaccard Index overlap analysis and clustering

We used the R package GenometriCorr v1.1.17 (http://genometricorr.sourceforge.net/)^110^ to calculate the Jaccard Index (JI), a measure of correlation between two genomic intervals, for ChIP-seq and ATAC-seq peaks and differential sites (up and down, only those >100 in number). Clustering was performed with the ‘Heatmap’ function of the Bioconductor package ‘ComplexHeatmap’.

### Comparison of synchronised versus unsynchronised differentiation by RNA-seq

Raw fastq datasets were downloaded from ENA (https://www.ebi.ac.uk/ena) and mapped to GRCh38 human genome reference assembly using STAR v2.6.1^111^. Differential expression analysis was performed with DESeq2^81^, and genes with absolute log_2_ FC ⋝2 and adjusted *P* ⋜ 0.001 were deemed as differentially expressed.

### Data visualisation

The box plots depicted in Supplementary Figs. 1k, m; 5e, g, h; 6e represent the smallest value and largest value as the box size, with the mean shown inside the box as a line.

### Statistics and reproducibility

Experiments were performed in three independent biological repeats unless stated otherwise. No data were excluded from the analyses. Statistical analysis for Supplementary Figs. 1e–g, k, m; 3d, e; 5d, e; 5g, h was performed by two-way ANOVA with multiple comparisons with Tukey correction and **** marks adjusted *P* value <0.0001, *** is adjusted *P* value <0.001, ** is adjusted *P* value <0.01, * is adjusted *P* value <0.05.

### Reporting summary

Further information on research design is available in the Nature Portfolio Reporting Summary linked to this article.

## Supplementary information

Supplementary Information

Description of Additional Supplementary Files

Supplementary Data 1

Supplementary Data 2

Reporting Summary

## Data Availability

Processed genome-wide datasets are publicly accessible in a genome browser at http://ngs.sanger.ac.uk/production/endoderm. Raw ChIP-seq data collected at 0, 24, 48 and 72 h is available at GEO DataSets under accession number PRJNA593217. Raw ChIP-seq for 12 and 36 h, ATAC-seq and RNA-seq data were publicly available in the ArrayExpress collection of BioStudies under accession numbers E-MTAB-9276, E-MTAB-9124 and E-MTAB-9194, respectively. Source data are provided with this paper. Statistical analyses are added with source data. Source data are provided with this paper.

## Source data

Source Data

## Code availability

The code used to analyse sequencing datasets is available at: https://github.com/pmb59/endoderm/ (10.5281/zenodo.7436993).
