# Supplementary material for: Epigenetic and transcriptional regulations prime cell fate before division during human pluripotent stem cell differentiation
Source: Nat Commun. 2023 Jan 25;14:405. doi: 10.1038/s41467-023-36116-9 (PMC9876972; doi:10.1038/s41467-023-36116-9)
Supplement: Supplementary file 1 — Supplementary Information [file 41467_2023_36116_MOESM1_ESM.pdf]

Supplementary Information

Epigenetic and transcriptional regulations prime cell fate before division during human pluripotent stem cell differentiation

Pedro Madrigal, Siwei Deng, Yuliang Feng, Stefania Militi, Kim Jee Goh, Reshma Nibhani,

Rodrigo Grandy, Anna Osnato, Daniel Ortmann, Stephanie Brown, Siim Pauklin

Supplementary Table 1. Antibodies.

All primary antibodies were used at 1:1000 dilution for western blotting, at 1:100 dilution for Immunofluorescence microscopy and flow cytometry, and 10μg per sample for ChIP unless otherwise specified. Secondary antibodies were used at 1:10,000 dilution for western blotting, and at 1:1000 for Immunofluorescence microscopy and flow cytometry unless otherwise specified.

Antibody raised against	Catalog number	Company	Clone/Lot
Histone H3	ab1791	Abcam	Poly
Histone H3 (tri methyl K4)	ab8580	Abcam	Poly
Histone H3 (tri methyl K27)	C15200181 (MAb-181-050)	Diagenode	001-13
Histone H3 (mono methyl K4)	ab8895	Abcam	Poly
Histone H3 (acetyl K27)	ab4729	Abcam	Poly

Histone H3 (tri methyl K36)	ab9050	Abcam	Poly
Actin, clone C4	MAB1501	Chemicon	AC-74
Brachyury (T)	af2085	R&D Systems	Poly
EOMES	ab23345	Abcam	Poly
Nanog	af1997	R&D Systems	Poly
Nestin (Rat-401)	sc-33677	Santa Cruz	RAT-401
Oct-3/4 (C-10)	sc-5279	Santa Cruz	C-10
Pax6	PRB-278P-100	Covance	Poly
Sox1	AF3369	R&D Systems	Poly
Sox17	AF1924	R&D Systems	Poly
Sox2	AF2018	R&D Systems	Poly
CXCR4	MAB173	R&D Systems	44717
Tra-1-60	sc-21705	Santa Cruz	
FRA2 (D2F1E)	19967S	Cell Signaling Technology	D2F1E
JUN	22114-1-AP	Proteintech	Poly
c-JUN (60A8)	9165T	Cell Signaling Technology	60A8
SMAD2/3	AF3797	Bio-Techne	Poly
GATA4	104604-T36	Sino Biological	Poly
PDX1	AF2419	Bio-Techne	Poly
SOX9	ab185230	AbCam	EPR14335
APC anti-human CD142 [NY2]	365205	Biolegend	NY2

Alexa Fluor 647 goat α-mouse IgM	A21238	Invitrogen	Poly
Alexa Fluor 647 donkey α-mouse IgG	A31571	Invitrogen	Poly
Alexa Fluor 647 donkey α-goat	A21447	Invitrogen	Poly

Supplementary Table 2. QPCR Primers.

**Gene**	**Forward sequence (5' to 3')**	**Reverse sequence (5' to 3')**
ACTB	CTGGAACGGTGAAGGTGACA	AAGGGACTTCCTGTAACAATGCA
BRACHYURY (T)	TGCTTCCCTGAGACCCAGTT	GATCACTTCTTTCCTTTGCATCAAG
CDX2	GGCAGCCAAGTGAAAACCAG	TTCCTCTCCTTTGCTCTGCG
EOMES	ATCATTACGAAACAGGGCAGGC	CGGGGTTGGTATTTGTGTAAGG
FOXA2	GGGAGCGGTGAAGATGGA	TCATGTTGCTCACGGAGGAGTA
GATA4	TCCCTCTTCCCTCCTCAAAT	TCAGCGTGTAAAGGCATCTG
HAND1	GTGCGTCCTTTAATCCTCTTC	GTGAGAGCAAGCGGAAAAG
MIXL1	GGTACCCCGACATCCACTTG	TAATCTCCGGCCTAGCCAAA
NANOG	CATGAGTGTGGATCCAGCTTG	CCTGAATAAGCAGATCCATGG
NKX2.5	GAGCCGAAAAGAAAGCCTGAA	CACCGACACGTCTCACTCAG
POU5F1	AGTGAGAGGCAACCTGGAGA	ACACTCGGACCACATCCTTC
SOX17	CGCACGGAATTTGAACAGTA	GGATCAGGGACCTGTCACAC
SOX2	TGGACAGTTACGCGCACAT	CGAGTAGGACATGCTGTAGGT

Supplementary Table 3. ChIP Primers.

**Primer name**	**Experimental usage**	**Sequence**
MESP2:90326940 CHIP F	Additional binding sites	AGGGACAGAGCACCATCATC

MESP2:90326940 CHIP R	Additional binding sites	ACTTAGCAAATGGCCTGACG
MESP2:90319794 CHIP F	Additional binding sites	CCTCCTCCTCCGATTCGT
MESP2:90319794 CHIP R	Additional binding sites	CAAGGAGGGAGGCAGAAAG
MESP2:90303697 CHIP F	Smad2/3 / FOSL2 / JUN ChIP	CCCTCACACCTTCAGCTCTC
MESP2:90303697 CHIP R	Smad2/3 / FOSL2 / JUN ChIP	TTCCTGGAATCTGCCAAGTC
MESP1:90294197 CHIP F	Additional binding sites	CTCGATCTTGGTCAGGCTCT
MESP1:90294197 CHIP R	Additional binding sites	CCAGTGAGCGGGAGAAACT
MESP1:90291241 CHIP F	Smad2/3 / FOSL2 / JUN ChIP	GTCCCCTGCCTATCTCCAGT
MESP1:90291241 CHIP R	Smad2/3 / FOSL2 / JUN ChIP	CATTGGCTAGCCCCCTTC
CER1:14722865 CHIP F	Smad2/3 / FOSL2 / JUN ChIP	GAAGGCAGGTGGTCAGTAGC
CER1:14722865 CHIP R	Smad2/3 / FOSL2 / JUN ChIP	TGCTCTTTAAGCCCCAGACA
CER1:14723566 CHIP F	Additional binding sites	CATGTTCCTCAACAGGATACCA
CER1:14723566 CHIP R	Additional binding sites	CCAGCGTCACACACAAAGTT
EOMES:27755748 CHIP F	Smad2/3 / FOSL2 / JUN ChIP	TGGCGGAGAATGTAAACAAA
EOMES:27755748 CHIP R	Smad2/3 / FOSL2 / JUN ChIP	TGGCCCCAATTAACACCTTA
FOXA2:22559856 CHIP F	Smad2/3 / FOSL2 / JUN ChIP	TCCCCGACATCCTTTTTATG
FOXA2:22559856 CHIP R	Smad2/3 / FOSL2 / JUN ChIP	GGGACAATGGAGGCTGAAAG
CDX2:28536122 CHIP F	Additional binding sites	CAGGAGAGCCACGCATTC
CDX2:28536122 CHIP R	Additional binding sites	TTGCAAGCTGATTTGGAAAA
CDX2:28528528 CHIP F	Additional binding sites	TTTGAAAAGCTTGGCGTCTT
CDX2:28528528 CHIP R	Additional binding sites	CGGGATTCCCTGGAGAGAT
MIXL1:226411093 CHIP F	Additional binding sites	GATTTGACCCGGAGAAGAGA
MIXL1:226411093 CHIP R	Additional binding sites	AGGAGGGAGGCGTGAACTT

MIXL1:226398572 CHIP F	Smad2/3 / FOSL2 / JUN ChIP	AGCTGACCACTTCCTCTTGC
MIXL1:226398572 CHIP R	Smad2/3 / FOSL2 / JUN ChIP	CAGTTTTGCCAGCATTGAAA
GATA4:11567708 CHIP F	Smad2/3 / FOSL2 / JUN ChIP	TGAAGCGCTTTTAAATTGTCC
GATA4:11567708 CHIP R	Smad2/3 / FOSL2 / JUN ChIP	GTCTGGCTCCCCCAAGAG
LZTS1:20092281 CHIP F	Smad2/3 / FOSL2 / JUN ChIP	TAATTCCCCCTCCCTGCTAA
LZTS1:20092281 CHIP R	Smad2/3 / FOSL2 / JUN ChIP	CAGCCCTCCCAGAATAATCA
GSC:95241963 CHIP F	Smad2/3 / FOSL2 / JUN ChIP	AGGAATGTGGATTGGACTGC
GSC:95241963 CHIP R	Smad2/3 / FOSL2 / JUN ChIP	GGCAAGACTGTGAGAGCAGA
GSC:95229907 CHIP F	Additional binding sites	AAGGTGCATTCCTCAATTCC
GSC:95229907 CHIP R	Additional binding sites	GATTCCTTCCTCACCTGCAA

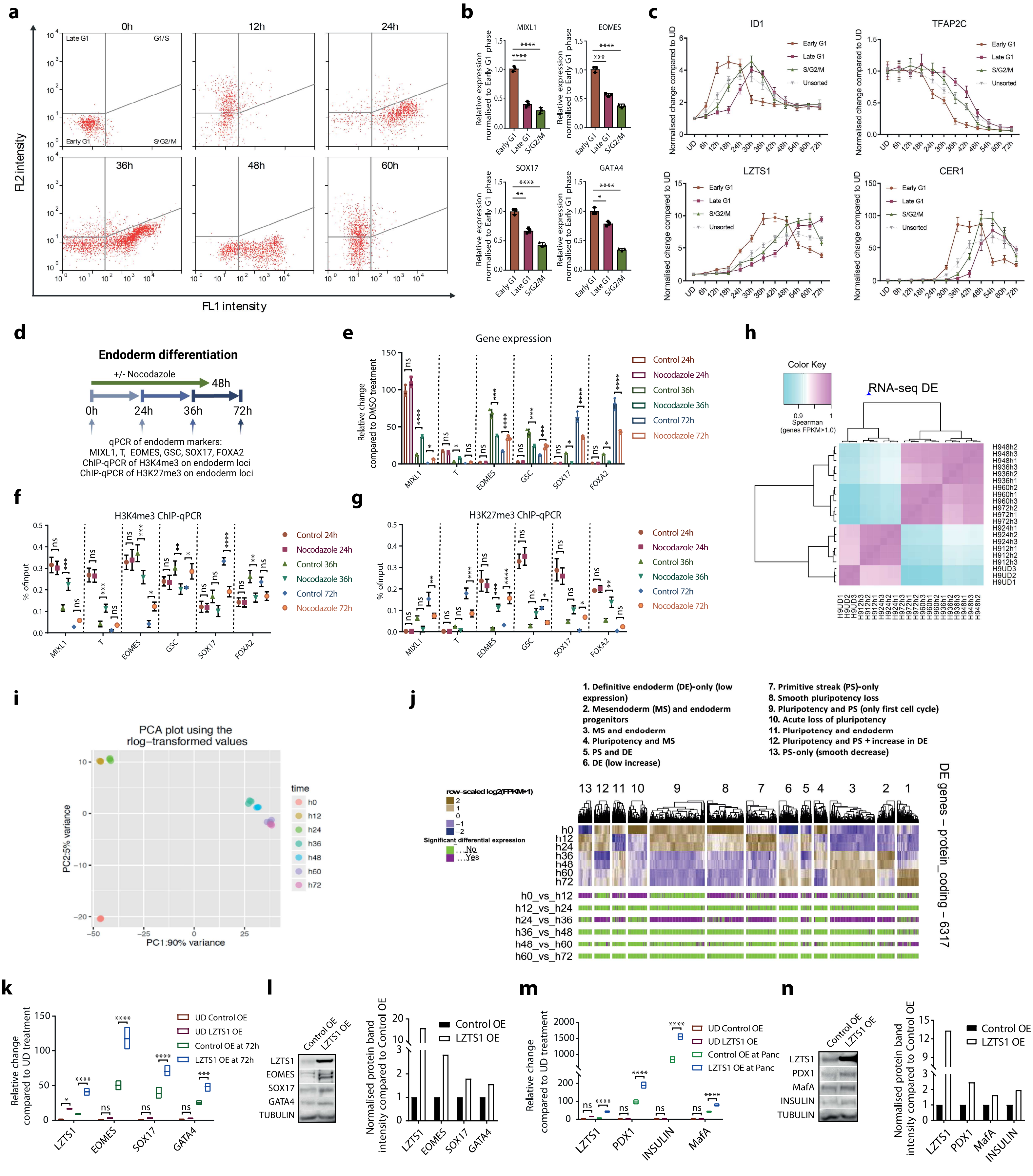

Supplementary Figure 1: Differentiation of cell cycle synchronised hESCs reveals novel transiently expressed early mesendoderm genes.
(a) FACS analyses showing cell cycle progression of EG1-FUCCI hPSCs differentiating into endoderm. (b) Expression of endoderm markers in different cell cycle phases of undifferentiated hESCs. Statistical analysis was performed by one-way ANOVA with Dunnett's multiple comparisons test. (c) H3K27ac ChIP-qPCR of ID1, TFAP2C, LZTS1, CER1 regulatory regions. FUCCI-hPSCs cells were sorted to early G1, late G1, S/G2/M or left unsorted, then differentiated to endoderm and collected for ChIP at different time points. Data in (b) and (c) depicts n = 3 biologically independent experiments. (d) Schematic representation of Nocodazole treatment and sample collection upon endoderm differentiation. (e) Gene expression analysis during the blocking of cell divisions in endoderm differentiation. (f-g) H3K4me3 ChIP-qPCR and H3K27me3 ChIP-qPCR at endoderm markers upon Nocodazole treatment. Data in (e) to (g) depicts n = 3 biologically independent experiments. (h) Hierarchical clustering analysis of Spearman correlation coefficients for gene expression in RNA-seq samples. (i) Principal component analysis (PCA) of G1 synchronised FUCCI-hPSCs differentiating into endoderm. (j) k-means clustering of differentially expressed genes (0 h, 12 h, 24 h, 36 h, 48 h, 60 h and 72 h) and annotated clusters (k=13). Model based optimal number of clusters with 6,317 protein coding genes was computed. The number of clusters k was selected as the one that minimised the Bayesian Information Criterion. (k-l) The effect of LZTS1 overexpression on endoderm marker expression analysed by (k) qPCR and (l) western blot. (m-n) The effect of LZTS1 overexpression on pancreatic beta cell marker expression analysed by (m) qPCR and (n) western blot. The bounds of box plots in (k and m) represent minima, maxima, and the horizontal line represents the mean of experiments. Statistical analysis in (e, f, g, k, m) was performed by two-way ANOVA with Tukey's multiple comparisons tests and * marks adjusted P-value <0.05 and ** marks adjusted P-value <0.01, *** marks adjusted P-value <0.001, **** marks adjusted P-value <0.0001. Data are presented as mean values +/- SD. Source data are provided as a Source Data file.

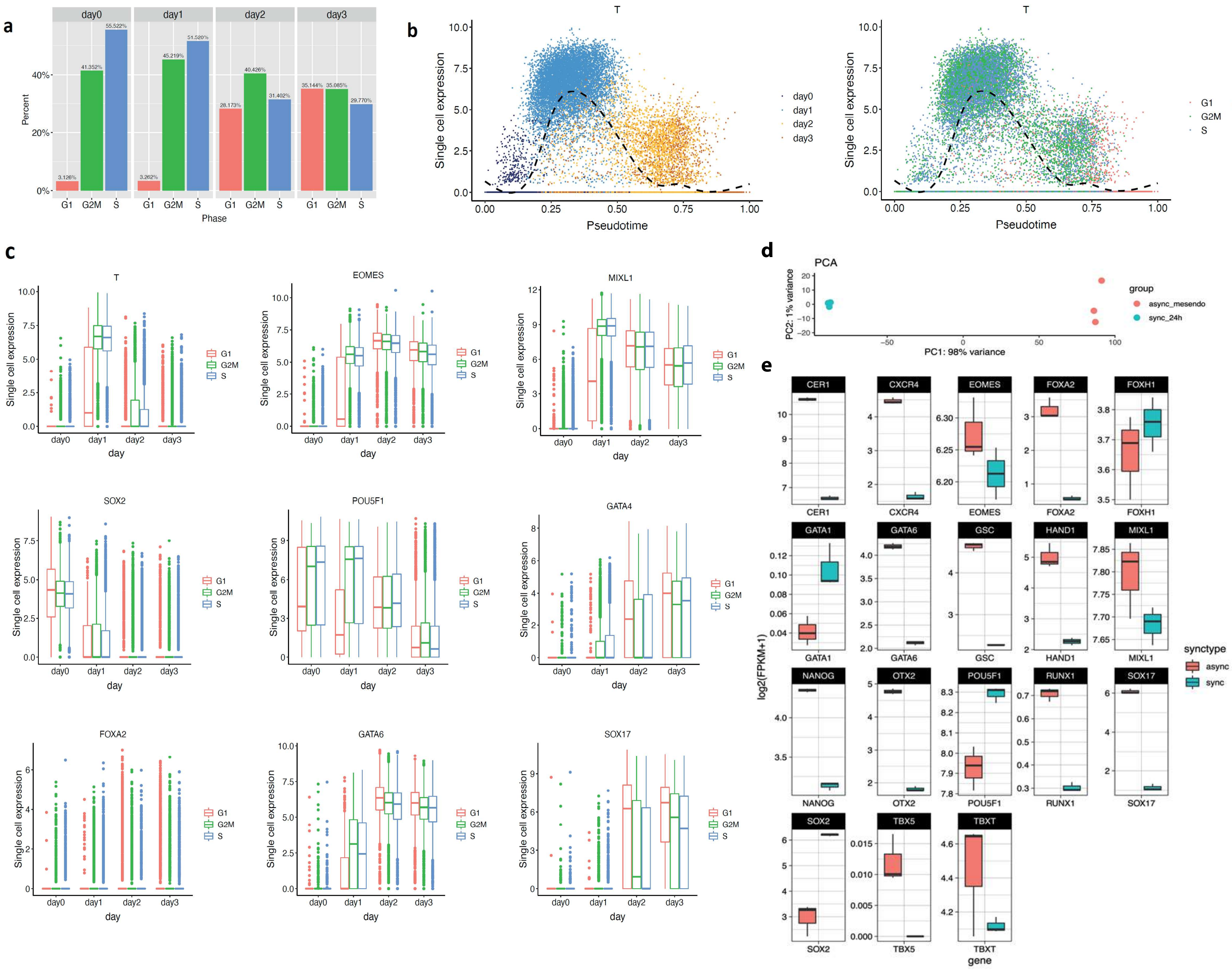

Supplementary Figure 2: Cell cycle phase assignment to a single-cell RNA sequencing dataset (36,044 cells) of asynchronous endoderm differentiation.
(a) Percentage of cells assigned to each cell cycle phase. (b) Single cell expression (y-axis) of mesendoderm marker T, plotted along pseudotime (x-axis). The black dashed line indicates the smoothed mean. (c) Boxplots representing the distribution of gene expression values of selected markers in all cells for each developmental stage in each assigned cell cycle phase. Gene expression values correspond to log2(Counts Per Million +1). The boxes show the interquartile range, with the median marked as heavy horizontal band. Whiskers represent the highest (lowest) datapoint within 1.5 times the interquartile range of the 75th (25th) percentile. Outliers are plotted separately as individual points beyond the whiskers on the boxplot. (d) Principal Component Analysis of synchronised and unsynchronised differentiation at 24 hours. Cells were analysed by bulk RNA-seq. Unsynchronised RNA-seq data was obtained from Valcourt et al. 42 (e) Comparative expression of marker genes in unsynchronised and synchronised cells at 24 hours of endodermal differentiation. The boxes show the interquartile range, with the median marked as heavy horizontal band. Whiskers represent the highest (lowest) datapoint within 1.5 times the interquartile range of the 75th (25th) percentile. Data in (c) and (e) depicts n = 3 biologically independent samples over an independent experiment.

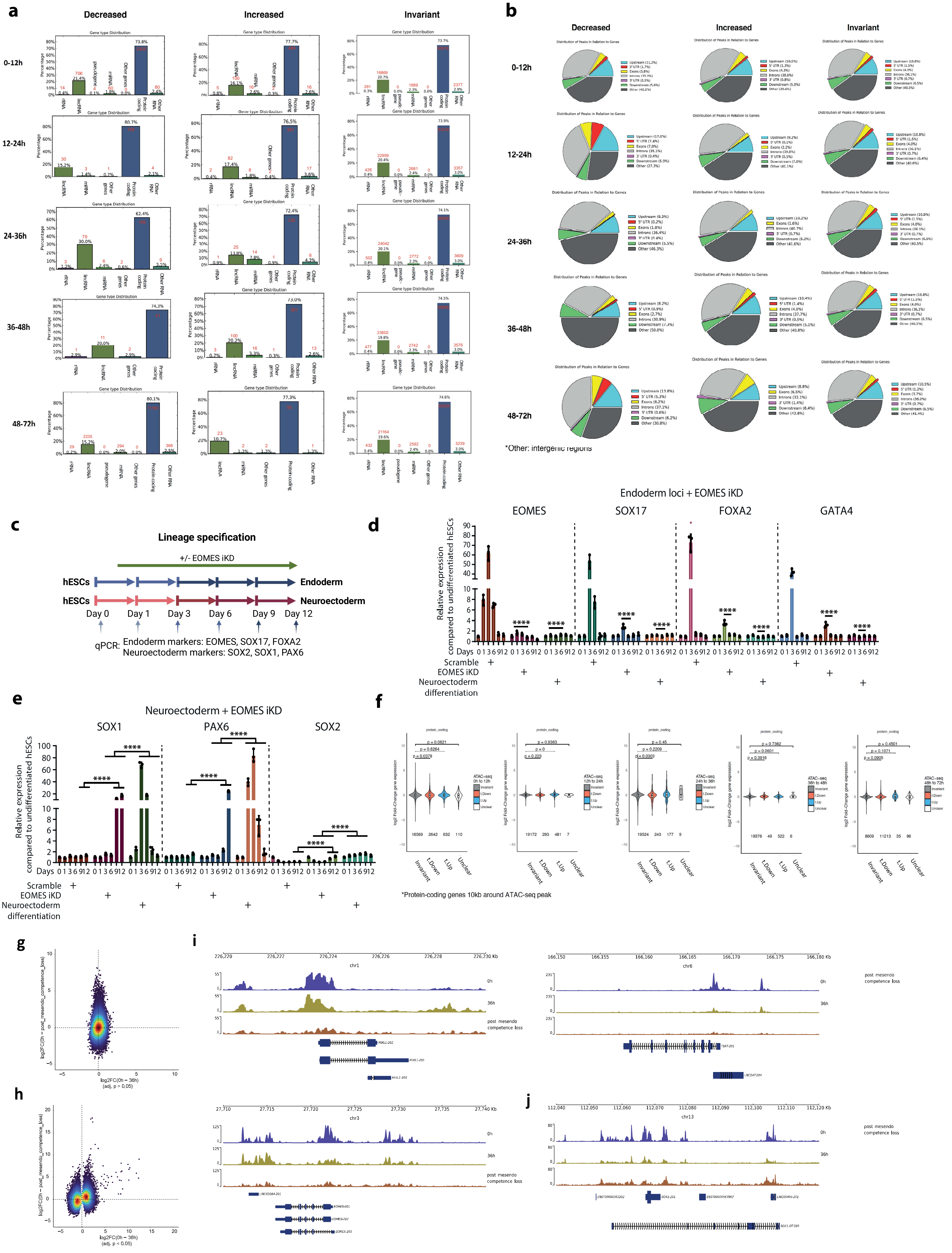
a
Decreased
Increased
Invariant
Gene type Distribution
0-12h
12-24h
24-36h
36-48h
48-72h
Percentage
rRNA
lincRNA
pseudogene
miRNA
Other genes
Protein coding
Other RNA
b
Decreased
Increased
Invariant
Distribution of Peaks in Relation to Genes
*Other: intergenic regions
c
Lineage specification
+/- EOMES iKD
hESCs
Endoderm
hESCs
Neuroectoderm
Day 0
Day 1
Day 3
Day 6
Day 9
Day 12
qPCR: Endoderm markers: EOMES, SOX17, FOXA2
Neuroectoderm markers: SOX2, SOX1, PAX6
d
Endoderm loci + EOMES iKD
EOMES
SOX17
FOXA2
GATA4
Relative expression compared to undifferentiated hESCs
Days
Scramble
EOMES iKD
Neuroectoderm differentiation
e
Neuroectoderm + EOMES iKD
SOX1
PAX6
SOX2
Relative expression compared to undifferentiated hESCs
Days
Scramble
EOMES iKD
Neuroectoderm differentiation
f
protein_coding
log2 Fold-Change gene expression
ATAC-seq
Invariant
t.Down
t.Up
Unclear
*Protein-coding genes 10kb around ATAC-seq peak
g
log2FC(0h – post_mesendo_competence_loss)
log2FC(0h – 36h)
(adj. p > 0.05)
h
log2FC(0h – post_mesendo_competence_loss)
log2FC(0h – 36h)
(adj. p < 0.05)
i
chr1
chr6
0h
36h
post mesendo competence loss
MIXL1-202
MIXL1-201
MIXL1-203
TBXT-201
LINC02547-204
j
chr3
chr13
EOMES-201
EOMES-202
EOMES-203
SOX1-201
SOX1-OT-203

Supplementary Figure 3: Characterization of genomic regions displaying changes in chromatin accessibility during differentiation.

(a) Annotation of chromatin accessibility peaks to gene category during differentiation of cell synchronised EG1-FUCCI hPSCs. Numbers in red colour represent the number of regions. (b) Annotation of chromatin accessibility peaks to intra- or inter-genic regions. (c) Schematic representation of the lineage specification experiment for analysing the effects of inducible EOMES knockdown on hESC differentiation. (d-e) The effects of EOMES knockdown on endoderm marker expression (d) and neuroectoderm marker expression (e). (f) Distribution of log2 fold-changes in RNA expression in regions with decreased, increased, or invariant chromatin accessibility defined by ATAC-seq. P-values reported by non-parametric two-sided Wilcoxon Rank Sum test. Analysis performed on protein-coding genes located 10 kb around ATAC-seq peaks. The distribution is shown as a violin plot wrapping a boxplot, where the median of the data is marked as a heavy horizontal line and the box indicates the interquartile range. Data in (d) to (f) depicts n = 3 biologically independent samples over an independent experiment. (g) Dot plot of genomic regions which were not changed significantly ($P > 0.05$) in the 0h undifferentiated cells compared to endoderm differentiated cells at 36h or neuroectoderm cells/post mesendo competence loss. X- and Y-axis are in log2 scale. (h) Dot plot of genomic regions which were changed significantly ($P < 0.05$) in the 0h undifferentiated cells compared to endoderm differentiated cells at 36h or neuroectoderm cells/ post mesendo competence loss. X- and Y-axis are in log2 scale. (i) Genomic regions of MIXL1, T and EOMES loci showing genome accessibility by ATAC-seq data visualisation. (j) The genomic region of the SOX1 locus shows genome accessibility by ATAC-seq data visualisation. Statistical analysis in (d, e) was performed by two-way ANOVA with Tukey's multiple comparisons tests and * marks adjusted P-value < 0.05 and ** marks adjusted P-value < 0.01, *** marks adjusted P-value < 0.001, **** marks adjusted P-value < 0.0001. Data are presented as mean values +/- SD. Source data are provided as a Source Data file.

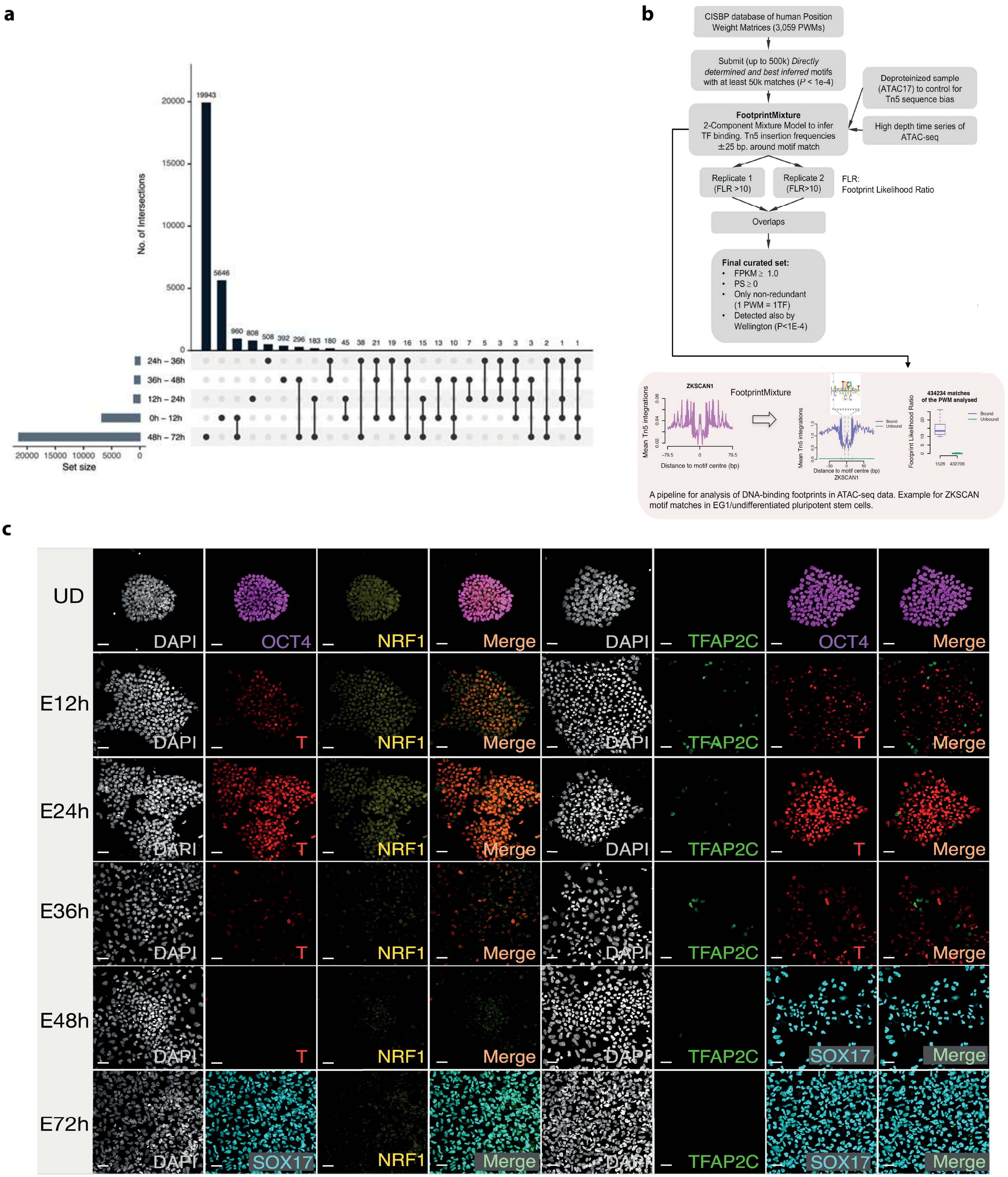

Supplementary Figure 4: Transcription factor footprinting analysis in chromatin accessibility regions during endoderm differentiation and immunofluorescence microscopy.

(a) Overlap analysis of differential chromatin accessibility regions between timepoints during endoderm differentiation. (b) Computational pipeline for digital genomic footprinting in ATAC-seq data to identify transcription factors footprints during endoderm differentiation. Example for ZKSCAN1 motif matches for CisBP Position Weight Matrix M4646 at 0 h. (c) Immunofluorescence microscopy staining of OCT4, NRF1, T, TFAP2C and SOX17 in synchronised hESCs during endoderm differentiation. The scale bar represents 100µm.

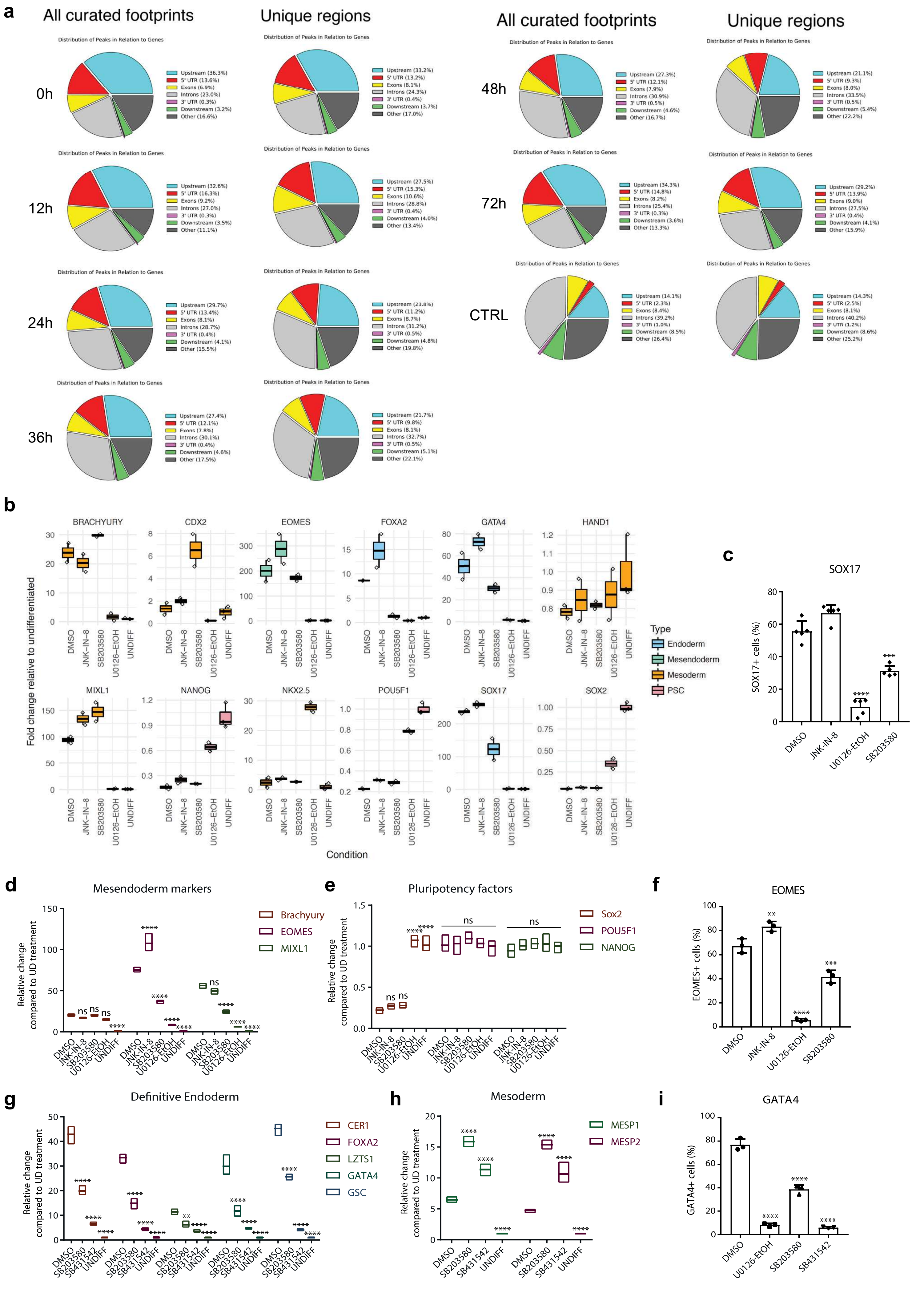

a
All curated footprints
Unique regions
Distribution of Peaks in Relation to Genes
0h
Upstream (36.3%)
5' UTR (13.6%)
Exons (6.9%)
Introns (23.0%)
3' UTR (0.3%)
Downstream (3.2%)
Other (16.6%)
Upstream (33.2%)
5' UTR (13.2%)
Exons (8.1%)
Introns (24.3%)
3' UTR (0.4%)
Downstream (3.7%)
Other (17.0%)
12h
Upstream (32.6%)
5' UTR (16.3%)
Exons (9.2%)
Introns (27.0%)
3' UTR (0.3%)
Downstream (3.5%)
Other (11.1%)
Upstream (27.5%)
5' UTR (15.3%)
Exons (10.6%)
Introns (28.8%)
3' UTR (0.4%)
Downstream (4.0%)
Other (13.4%)
24h
Upstream (29.7%)
5' UTR (13.4%)
Exons (8.1%)
Introns (28.7%)
3' UTR (0.4%)
Downstream (4.1%)
Other (15.5%)
Upstream (23.8%)
5' UTR (11.2%)
Exons (8.7%)
Introns (31.2%)
3' UTR (0.5%)
Downstream (4.8%)
Other (19.8%)
36h
Upstream (27.4%)
5' UTR (12.1%)
Exons (7.8%)
Introns (30.1%)
3' UTR (0.4%)
Downstream (4.6%)
Other (17.5%)
Upstream (21.7%)
5' UTR (9.8%)
Exons (8.1%)
Introns (32.7%)
3' UTR (0.5%)
Downstream (5.1%)
Other (22.1%)
48h
Upstream (27.3%)
5' UTR (12.1%)
Exons (7.9%)
Introns (30.9%)
3' UTR (0.5%)
Downstream (4.6%)
Other (16.7%)
Upstream (21.1%)
5' UTR (9.3%)
Exons (8.0%)
Introns (33.5%)
3' UTR (0.5%)
Downstream (5.4%)
Other (22.2%)
72h
Upstream (34.3%)
5' UTR (14.8%)
Exons (8.2%)
Introns (25.4%)
3' UTR (0.3%)
Downstream (3.6%)
Other (13.3%)
Upstream (29.2%)
5' UTR (13.9%)
Exons (9.0%)
Introns (27.5%)
3' UTR (0.4%)
Downstream (4.1%)
Other (15.9%)
CTRL
Upstream (14.1%)
5' UTR (2.3%)
Exons (8.4%)
Introns (39.2%)
3' UTR (1.0%)
Downstream (8.5%)
Other (26.4%)
Upstream (14.3%)
5' UTR (2.5%)
Exons (8.1%)
Introns (40.2%)
3' UTR (1.2%)
Downstream (8.6%)
Other (25.2%)
b
BRACHYURY
CDX2
EOMES
FOXA2
GATA4
HAND1
MIXL1
NANOG
NKX2.5
POU5F1
SOX17
SOX2
Fold change relative to undifferentiated
DMSO
JNK-IN-8
SB203580
U0126-EtOH
UNDIFF
Condition
Type
Endoderm
Mesendoderm
Mesoderm
PSC
c
SOX17
SOX17+ cells (%)
DMSO
JNK-IN-8
U0126-EtOH
SB203580
d
Mesendoderm markers
Relative change compared to UD treatment
Brachyury
EOMES
MIXL1
e
Pluripotency factors
Sox2
POU5F1
NANOG
f
EOMES
EOMES+ cells (%)
g
Definitive Endoderm
CER1
FOXA2
LZTS1
GATA4
GSC
SB431542
h
Mesoderm
MESP1
MESP2
i
GATA4
GATA4+ cells (%)

Supplementary Figure 5: Transcription factor binding footprint characterisation and experimental work with pathway inhibitors.
(a) Annotation of transcription factor footprints into intra- or inter-genic regions. A deproteinised (naked DNA) ATAC-seq sample was used a control. (b) Q-PCR analyses showing the effect of different inhibitors of AP1-related signalling pathways on the expression of pluripotency (NANOG, POU5F1/OCT4 and SOX2), mesoderm (BRACHYURY/T, CDX2, NKX2.5, HAND1, MIXL1) and endoderm markers (FOXA2, SOX17, GATA4, EOMES). cA1ATD hPSC line was grown for 3 days in culture conditions inducing endoderm differentiation in the presence of DMSO (control), JNK-in-8, SB 203580, U0126. cA1ATD hPSCs grown in culture condition maintaining pluripotency were used as negative control. For Q-PCR, the boxes show the interquartile range, with the median marked as heavy horizontal band. Whiskers represent the highest (lowest) datapoint within 1.5 times the interquartile range of the 75th (25th) percentile. The diamonds represent each data point. (c) FACS analyses showing the expression of the endoderm marker SOX17 in cA1ATD hPSCs differentiated into endoderm in the presence of the denoted inhibitors. (d-e) The impact of JNK-in-8, SB 203580, and U0126-EtOH on the expression of (d) mesendoderm markers and (e) pluripotency markers at 24 h of endoderm differentiation. (f) Flow cytometry analysis of EOMES expression upon the treatment with JNK-in-8, SB 203580, and U0126. (g-h) The impact of SB 203580 and SB431542 on the expression of (g) definitive endoderm markers and (h) mesoderm markers at 36 h of endoderm differentiation. (i) Flow cytometry analysis of GATA4 expression upon the treatment with UO126-EtOH, SB 203580, and U0126-EtOH. The bounds of box plots in (d, e, g and h) represent minima, maxima, and the horizontal line represents the mean of experiments. One-way ANOVA in (c, f, i) was performed followed by Dunnett's multiple comparisons test where each of the 3 treatment conditions was compared against the control. Statistical analysis in (d, e, g, h) was performed by two-way ANOVA with Tukey's multiple comparisons tests and * marks adjusted P-value < 0.05 and ** marks adjusted P-value < 0.01, *** marks adjusted P-value < 0.001, **** marks adjusted P-value < 0.0001. Data in (c) to (i) depicts n = 3 biologically independent experiment. Data are presented as mean values +/- SD. Source data are provided as a Source Data file. Specific P-values shown in figures are listed in the Source Data file.

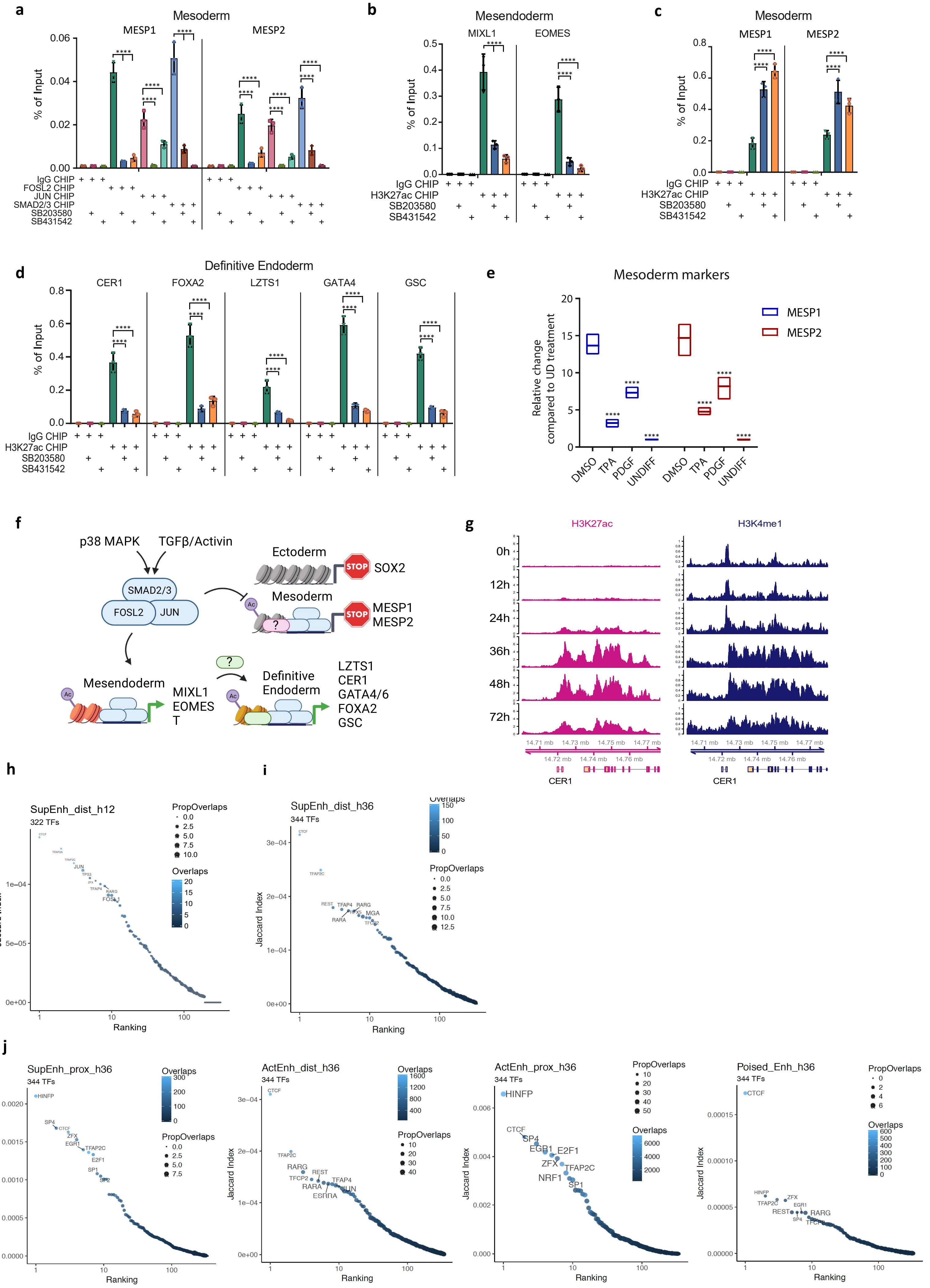
a
Mesoderm
MESP1
MESP2
% of Input
IgG CHIP
FOSL2 CHIP
JUN CHIP
SMAD2/3 CHIP
SB203580
SB431542
b
Mesendoderm
MIXL1
EOMES
IgG CHIP
H3K27ac CHIP
SB203580
SB431542
c
Mesoderm
MESP1
MESP2
d
Definitive Endoderm
CER1
FOXA2
LZTS1
GATA4
GSC
e
Mesoderm markers
Relative change compared to UD treatment
MESP1
MESP2
DMSO
TPA
PDGF
UNDIFF
f
p38 MAPK
TGFβ/Activin
SMAD2/3
FOSL2
JUN
Ectoderm
SOX2
Mesoderm
MESP1
MESP2
Mesendoderm
MIXL1
EOMES
T
Definitive Endoderm
LZTS1
CER1
GATA4/6
FOXA2
GSC
STOP
Ac
?
g
H3K27ac
H3K4me1
0h
12h
24h
36h
48h
72h
CER1
h
SupEnh_dist_h12
322 TFs
PropOverlaps
Overlaps
Jaccard Index
Ranking
i
SupEnh_dist_h36
344 TFs
j
SupEnh_prox_h36
ActEnh_dist_h36
ActEnh_prox_h36
Poised_Enh_h36
344 TFs
CTCF
HINFP
JUN
FOSL1
TFAP2C
TFAP4
RARG
REST
SP4
ZFX
EGR1
E2F1
SP1
NRF1

Supplementary Figure 6: p38-MAPK and TGFβ/Activin A signalling co-regulate AP-1 TFs and SMAD2/3 binding to germ layer loci at different stages of endoderm differentiation.

(a) p38-MAPK and TGFβ/Activin A signalling co-regulate AP-1 TFs FOSL2/JUN and SMAD2/3 binding to mesoderm genes MESP1 and MESP2 at 36 h time point after initiating endoderm differentiation. Early G1 phase synchronised cells were differentiated to endoderm for 36 h and treated with p38 MAPK and TGFβ/Activin A inhibitors for 12h as shown in Fig. 4g, followed by ChIP-qPCR. (b-d) p38-MAPK and TGFβ/Activin A signalling inhibition lead to the loss of H3K27ac modifications on regulatory regions of (b) primitive streak/mesendoderm genes MIXL1 and EOMES, (c) mesoderm genes MESP1 and MESP2, and (d) definitive endoderm genes CER1, FOXA2, LZTS1, GATA4, GSC. Statistical analysis was performed by 2-way ANOVA with multiple comparisons and **** marks adjusted P-value < 0.0001. (e) The effects of TPA and PDGF on mesoderm marker MESP1 and MESP2 expression in embryoid body formation conditions. The bounds of box plots in (e) represent minima, maxima, and the horizontal line represents the mean of experiments. Statistical analysis in (a-e) was performed by two-way ANOVA with Tukey's multiple comparisons tests and * marks adjusted P-value < 0.05 and ** marks adjusted P-value < 0.01, *** marks adjusted P-value < 0.001, **** marks adjusted P-value < 0.0001. Data in (a) to (e) depicts n = 3 biologically independent experiments. Data are presented as mean values +/- SD. (f) Schematic representation of the role of p38 MAPK-AP1 and TGFβ/Activin A-SMAD2/3 signalling pathways in regulating the specification of definitive endoderm at multiple differentiation stages. p38 MAPK and TGFβ/Activin A cooperate in signalling to SMAD2/3 and AP-1 TFs FOSL2/JUN which form an activative transcriptional complex (light blue) on mesendoderm loci. The complex induces activative histone modifications (H3K27ac, purple circle) at the key transcriptional regulator loci MIXL1 and EOMES that direct cellular specification to the next step by gene expression changes. Following the cellular transition through the mesendoderm stage, the SMAD2/3 and FOSL2/JUN complex is involved in increasing H3K27ac and the expression of definitive endoderm transcription factors CER1, GATA4/6, FOXA2, GSC, and LZTS1. SMAD2/3 and FOSL2/JUN complex could cooperate with mesendoderm TFs such as EOMES (light green) that help to target SMAD2/3-FOSL2/JUN complex to definitive endoderm TFs loci and induce their expression by co-binding with GATA4 and other definitive endoderm factors. At this stage, SMAD2/3-FOSL2/JUN complex also participate in the transient repression of MESP1 and MESP2 loci which could involve additional transcriptional regulators (pink). This suggests a mechanism by which SMAD2/3 together with other transcription factors forms a feed-forward mechanism that directs gradual stem cell differentiation, and could help to explain molecular mechanisms that are involved in cell-autonomous formation of complex tissues. Created with BioRender.com. (g) Super-enhancers found close to CER1 locus. (h-i) Association of TF footprints with distal super enhancer regions. Jaccard Index, total number of footprint overlaps with the differential region (Overlaps), and percentage of footprints of a TF that overlap with this region (PropOverlaps) at 12h (h) and 36h (i). (j) Jaccard Index between FPs and proximal super enhancers, active distal super enhancers, active proximal enhancers, and poised enhancers at 36h. Data are presented as mean values +/- SD. Source data are provided as a Source Data file. Specific P-values shown in figures are listed in the Source Data file.

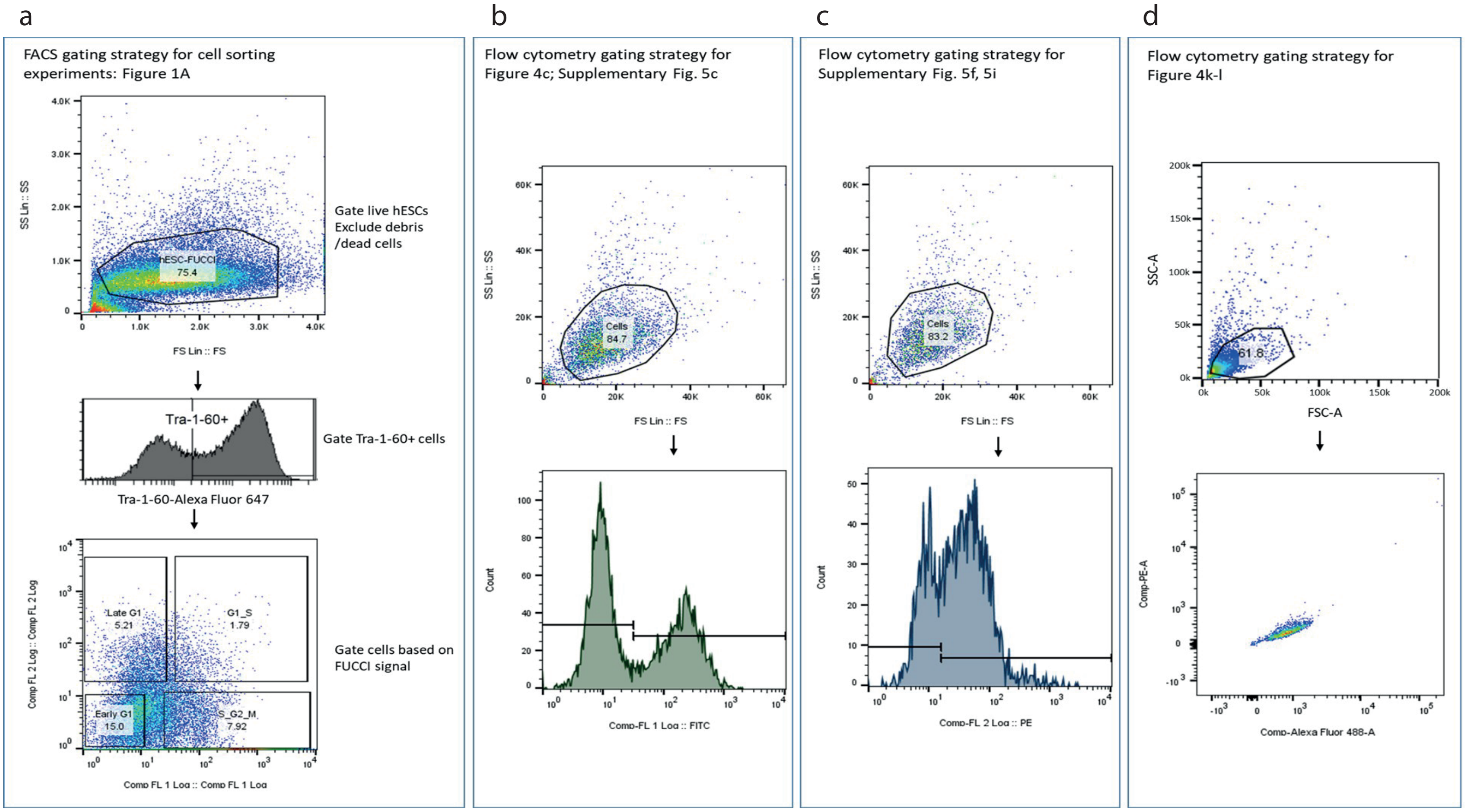

Supplementary Figure 7. Gating strategy for cell sorting and flow cytometry experiments.
(a) FACS gating strategy for cell sorting experiments for Figure 1A. (b) Flow cytometry gating strategy for Figure 4c and Supplementary Figure 5c. (c) Flow cytometry gating strategy for Supplementary Figure 5f and 5i. (d) Flow cytometry gating strategy for Figure 4k-l.
