# Supplementary material for: Epigenetic and transcriptional regulations prime cell fate before division during human pluripotent stem cell differentiation
Source: Nat Commun. 2023 Jan 25;14:405. doi: 10.1038/s41467-023-36116-9 (PMC9876972; doi:10.1038/s41467-023-36116-9)
Supplement: Supplementary file 2 — Description of Additional Supplementary Files [file 41467_2023_36116_MOESM2_ESM.pdf]

Description of Additional Supplementary Files

File Name: Supplementary Data 1
Description: Genes expressed in the different clusters identified by k-means clustering of differentially expressed genes during differentiation of EG1 FUCCI-hPSCs (0h, 12h, 24h, 36h, 48h, 60h and 72h) as denoted in Fig. 1h and Supplementary Fig. 1j.

File Name: Supplementary Data 2
Description: GO enrichment analysis in differential open chromatin regions.
