# Supplementary material for: Epigenetic and transcriptional regulations prime cell fate before division during human pluripotent stem cell differentiation
Source: Nat Commun. 2023 Jan 25;14:405. doi: 10.1038/s41467-023-36116-9 (PMC9876972; doi:10.1038/s41467-023-36116-9)
Supplement: Supplementary file 5 — Reporting Summary [file 41467_2023_36116_MOESM5_ESM.pdf]

nature portfolio

Corresponding author(s): Siim Pauklin

Last updated by author(s): 29/12/22

Reporting Summary

Nature Portfolio wishes to improve the reproducibility of the work that we publish. This form provides structure for consistency and transparency in reporting. For further information on Nature Portfolio policies, see our Editorial Policies and the Editorial Policy Checklist.

Statistics

For all statistical analyses, confirm that the following items are present in the figure legend, table legend, main text, or Methods section.

n/a	Confirmed	
☐	☒	The exact sample size (*n*) for each experimental group/condition, given as a discrete number and unit of measurement
☒	☐	A statement on whether measurements were taken from distinct samples or whether the same sample was measured repeatedly
☐	☒	The statistical test(s) used AND whether they are one- or two-sided<br>*Only common tests should be described solely by name; describe more complex techniques in the Methods section.*
☐	☒	A description of all covariates tested
☐	☒	A description of any assumptions or corrections, such as tests of normality and adjustment for multiple comparisons
☐	☒	A full description of the statistical parameters including central tendency (e.g. means) or other basic estimates (e.g. regression coefficient) AND variation (e.g. standard deviation) or associated estimates of uncertainty (e.g. confidence intervals)
☐	☒	For null hypothesis testing, the test statistic (e.g. *F*, *t*, *r*) with confidence intervals, effect sizes, degrees of freedom and *P* value noted<br>*Give P values as exact values whenever suitable.*
☒	☐	For Bayesian analysis, information on the choice of priors and Markov chain Monte Carlo settings
☒	☐	For hierarchical and complex designs, identification of the appropriate level for tests and full reporting of outcomes
☒	☐	Estimates of effect sizes (e.g. Cohen's *d*, Pearson's *r*), indicating how they were calculated

Our web collection on statistics for biologists contains articles on many of the points above.

Software and code

Policy information about availability of computer code

Data collection	No software was used for data collection.
Data analysis	The code used to analyse sequencing datasets is available at:<br>The code used to analyse sequencing datasets is available at: https://github.com/pmb59/endoderm/ (https://doi.org/10.5281/zenodo.7436993).

For manuscripts utilizing custom algorithms or software that are central to the research but not yet described in published literature, software must be made available to editors and reviewers. We strongly encourage code deposition in a community repository (e.g. GitHub). See the Nature Portfolio guidelines for submitting code & software for further information.

Data

Policy information about availability of data

All manuscripts must include a data availability statement. This statement should provide the following information, where applicable:

- Accession codes, unique identifiers, or web links for publicly available datasets
- A description of any restrictions on data availability
- For clinical datasets or third party data, please ensure that the statement adheres to our policy

Processed genome-wide datasets are publicly accessible in a genome browser at http://ngs.sanger.ac.uk/production/endoderm. Raw ChIP-seq data collected at 0 h, 24 h, 48 h, and 72 h is available at GEO DataSets (PRJNA593217). Raw ChIP-seq for 12 h and 36 h, ATAC-seq and RNA-seq data are publicly available in ArrayExpress

under accessions E-MTAB-9276, E-MTAB-9124 and E-MTAB-9194, respectively. Any databases/datasets used in the study are mentioned under the "Data availability" section in the manuscript.

Human research participants

Policy information about studies involving human research participants and Sex and Gender in Research.

Reporting on sex and gender	N/A
Population characteristics	N/A
Recruitment	N/A
Ethics oversight	N/A

Note that full information on the approval of the study protocol must also be provided in the manuscript.

Field-specific reporting

Please select the one below that is the best fit for your research. If you are not sure, read the appropriate sections before making your selection.

☒ Life sciences ☐ Behavioural & social sciences ☐ Ecological, evolutionary & environmental sciences

For a reference copy of the document with all sections, see nature.com/documents/nr-reporting-summary-flat.pdf

Life sciences study design

All studies must disclose on these points even when the disclosure is negative.

Sample size	We used H9 hESCs and two hIPSC lines as the main experimental models. The sample size follows accepted scientific standards in the research field, and includes representative cells for both hESCs and hIPSCs. No sample size calculation was performed. Experiments were performed in triplicate which follows general scientific standards in the research field.
Data exclusions	No data was excluded.
Replication	Experiments were performed in triplicate unless specified. All attempts at replication were successful.
Randomization	Randomization was not applicable to the study since it did not involve patients and study subjects. The relevant experimental controls were used in each experiment as described in the manuscript for investigating molecular effects.
Blinding	Blinding was not applicable to the study since it did not involve patients and clinical studies. The relevant experimental controls were used in each experiment as described in the manuscript for investigating molecular effects.

Reporting for specific materials, systems and methods

We require information from authors about some types of materials, experimental systems and methods used in many studies. Here, indicate whether each material, system or method listed is relevant to your study. If you are not sure if a list item applies to your research, read the appropriate section before selecting a response.

Materials & experimental systems

n/a	Involved in the study
☐	☒ Antibodies
☐	☒ Eukaryotic cell lines
☒	☐ Palaeontology and archaeology
☒	☐ Animals and other organisms
☒	☐ Clinical data
☒	☐ Dual use research of concern

Methods

n/a	Involved in the study
☐	☒ ChIP-seq
☐	☒ Flow cytometry
☒	☐ MRI-based neuroimaging

Antibodies

Antibodies used	Supplementary Table 3. Antibodies.<br>All primary antibodies were used at 1:1000 dilution for western blotting, at 1:100 dilution for Immunofluorescence microscopy and

flow cytometry, and 10µg per sample for ChIP unless otherwise specified. Secondary antibodies were used at 1:10,000 dilution for western blotting, and at 1:1000 for Immunofluorescence microscopy and flow cytometry unless otherwise specified.
Antibody raised against, Catalog number, Company, Clone/Lot
Histone H3 ab1791 Abcam Poly
Histone H3 (tri methyl K4) ab8580 Abcam Poly
Histone H3 (tri methyl K27) C15200181
(MAb-181-050) Diagenode 001-13
Histone H3 (mono methyl K4) ab8895 Abcam Poly
Histone H3 (acetyl K27) ab4729 Abcam Poly
Histone H3 (tri methyl K36) ab9050 Abcam Poly
Actin, clone C4 MAB1501 Chemicon AC-74
Brachyury (T) af2085 R&D Systems Poly
EOMES ab23345 Abcam Poly
Nanog af1997 R&D Systems Poly
Nestin (Rat-401) sc-33677 Santa Cruz RAT-401
Oct-3/4 (C-10) sc-5279 Santa Cruz C-10
Pax6 PRB-278P-100 Covance Poly
Sox1 AF3369 R&D Systems Poly
Sox17 AF1924 R&D Systems Poly
Sox2 AF2018 R&D Systems Poly
CXCR4 MAB173 R&D Systems 44717
Tra-1-60 sc-21705 Santa Cruz
FRA2 (D2F1E) 19967S Cell Signaling Technology D2F1E
JUN 22114-1-AP Proteintech Poly
c-JUN (60A8) 9165T Cell Signaling Technology 60A8
SMAD2/3 AF3797 Bio-Techne Poly
GATA4 104604-T36 Sino Biological Poly
PDX1 AF2419 Bio-Techne Poly
SOX9 ab185230 AbCam EPR14335
APC anti-human CD142 [NY2] 365205 Biolegend NY2
Alexa Fluor 647 goat α-mouse IgM A21238 Invitrogen Poly
Alexa Fluor 647 donkey α-mouse IgG A31571 Invitrogen Poly
Alexa Fluor 647 donkey α-goat A21447 Invitrogen Poly

Validation

We used only commercial antibodies which have all been validated by the company selling the antibody. The antibodies have been validated for specificity by using gene knockouts/knockdows and immunoprecipitation techniques as described by the companies selling the antibodies. Besides this, the antibodies have been further validated in publications citing the antibodies.

Eukaryotic cell lines

Policy information about cell lines and Sex and Gender in Research

Cell line source(s)

H9 from WiCell
hIPSC lines were obtained from the James and Lillian Martin Centre for Stem Cell Research and the Wellcome Sanger Institute.

Authentication

All cell lines have been authenticated by the providers by using cell morphology, Karyotyping, and PCR assays with species-specific primers as well as gene expression profiling.

Mycoplasma contamination

All cell lines tested negative for mycoplasma.

Commonly misidentified lines
(See ICLAC register)

No commonly misidentified lines were used in this study.

ChIP-seq

Data deposition

☒ Confirm that both raw and final processed data have been deposited in a public database such as GEO.

☒ Confirm that you have deposited or provided access to graph files (e.g. BED files) for the called peaks.

Data access links
May remain private before publication.

All the raw ChIP-seq datasets are available in FASTQ format from ArrayExpress/BioStudies
https://www.ebi.ac.uk/biostudies/arrayexpress/studies/E-MTAB-9276
and from GEO PRJNA593217
https://www.ncbi.nlm.nih.gov/bioproject/PRJNA593217/

Final processed data is available from a website hosted at the Wellcome Sanger Institute:
http://ngs.sanger.ac.uk/production/endoderm/
which include BED and bigWig files that can be visualized online in a genome browser.

Files in database submission

See the links above for access (many files)

Genome browser session
(e.g. UCSC)

http://ngs.sanger.ac.uk/production/endoderm
(with Biodalliance Genome Browser)

Methodology

Replicates	Two biological replicates per time point, per histone modification, except where indicated in Supplementary Methods.
Sequencing depth	Equimolar amounts of each library were pooled, and this multiplexed library was diluted to 8pM before sequencing using an Illumina HiSeq 2000 with 75 bp paired-end reads. More than 40bn reads were generated in total.
Antibodies	All antibodies used are commercially available and have been verified by the company to be suitable for ChIP-seq experiments.<br>Antibody raised against Catalog number Company<br>Histone H3 ab1791 Abcam<br>Histone H3 (tri methyl K4) ab8580 Abcam<br>Histone H3 (tri methyl K27) C15200181<br>(MAb-181-050) Diagenode<br>Histone H3 (mono methyl K4) ab8895 Abcam<br>Histone H3 (acetyl K27) ab4729 Abcam<br>Histone H3 (tri methyl K36) ab9050 Abcam
Peak calling parameters	tool peakranger 1.18 in modes ranger (H3K4me3, H3K27ac; '-l 316 -b 200 -q 0.05'), ccat (H3K27me3; '-l 316 --win_size 1000 --win_step 100 --min_count 70 --min_score 7 -q 0.05') and bcp (H3K4me1, H3K36me3; '-l 316'). Adjacent peak regions closer than 40 bp were merged using the BEDTools suite. Only the peaks that were reproducible at an FDR of ≤0.05 in two biological replicates were selected for further processing.
Data quality	Only reads with mapping quality score ≥10 and aligned to autosomal and sex chromosomes were kept for further processing.
Software	BWA, peakranger, BEDTools

Flow Cytometry

Plots

Confirm that:

☒ The axis labels state the marker and fluorochrome used (e.g. CD4-FITC).

☒ The axis scales are clearly visible. Include numbers along axes only for bottom left plot of group (a 'group' is an analysis of identical markers).

☒ All plots are contour plots with outliers or pseudocolor plots.

☒ A numerical value for number of cells or percentage (with statistics) is provided.

Methodology

Sample preparation	Single cell suspensions were prepared by incubation in Cell Dissociation Buffer (Gibco) for 10 minutes at 37° followed by gentle pipetting. Cells were fixed in 4% PFA for 20 min at 4°C. This was followed by permeabilization and blocking with 10% serum + 0.1% Triton X-100 in PBS for 30 min at RT and incubation with primary antibody in 1% serum + 0.1% Triton X-100 for 2h at 4°C. After washing the samples three times with PBS, they were incubated with a secondary antibody for 2h at 4°C, washed three times with PBS and analysed by flow cytometry. Flow-cytometry was performed using a Cyan ADP flow-cytometer and at least 20,000 events were recorded. Data was analysed by FlowJo software. Cell cycle distribution was analysed by Click-It EdU incorporation Kit (Invitrogen) according to manufacturer's guidelines.
Instrument	Cyan ADP flow-cytometer
Software	FlowJo
Cell population abundance	20,000 events were recorded, subpopulations are generally above 1%.
Gating strategy	Cell dublets were removed by gating, and the positive gating according to forward and side scatter was performed by selecting events that excluded events that were clearly outsider the normal cell size thus representing cell debris and non-cellular counts.

☒ Tick this box to confirm that a figure exemplifying the gating strategy is provided in the Supplementary Information.
